# Comprehensive characterization of early-programmed tumor microenvironment by tumor-associated macrophages reveals galectin-1 as an immune modulatory target in breast cancer

**DOI:** 10.7150/thno.88917

**Published:** 2024-01-01

**Authors:** Hyewon Chung, Park Gyu-mi, Yi Rang Na, Yun-Sang Lee, Hongyoon Choi, Seung Hyeok Seok

**Affiliations:** 1 Macrophage Lab, Department of Microbiology and Immunology, and Institute of Endemic Disease, Seoul National University College of Medicine, Seoul 110-799, South Korea.; 2 Institute of Endemic Diseases, Seoul National University Medical Research Center (SNUMRC), Seoul, Republic of Korea.; 3 Department of Biomedical Sciences and Seoul National University College of Medicine, Seoul, Republic of Korea.; 4 Transdisciplinary Department of Medicine and Advanced Technology, Seoul National University Hospital, Seoul, South Korea.; 5 Department of Nuclear Medicine, Seoul National University Hospital, Seoul, Republic of Korea.; 6 Department of Nuclear Medicine, Seoul National University College of Medicine, Seoul, Republic of Korea.; 7 Cancer Research Institute, Seoul National University, Seoul, South Korea.

## Abstract

**Background:** In recent years, there has been considerable interest in the therapeutic targeting of tumor-associated macrophages (TAMs) to modulate the tumor microenvironment (TME), resulting in antitumoral phenotypes. However, key mediators suitable for TAM-mediated remodeling of the TME remain poorly understood.

**Methods:** In this study, we used single-cell RNA sequencing analyses to analyze the landscape of the TME modulated by TAMs in terms of a protumor microenvironment during early tumor development.

**Results:** Our data revealed that the depletion of TAMs leads to a decreased epithelial-to-mesenchymal transition (EMT) signature in cancer cells and a distinct transcriptional state characterized by CD8^+^ T cell activation. Moreover, notable alterations in gene expression were observed upon the depletion of TAMs, identifying Galectin-1 (Gal-1) as a crucial molecular factor responsible for the observed effect. Gal-1 inhibition reversed immune suppression via the reinvigoration of CD8^+^ T cells, impairing tumor growth and potentiating immune checkpoint inhibitors in breast tumor models.

**Conclusion:** These results provide comprehensive insights into TAM-mediated early tumor microenvironments and reveal immune evasion mechanisms that can be targeted by Gal-1 to induce antitumor immune responses.

## Introduction

Immune cells are co-opted by cancer cells early in cancer development to establish a supportive tumor microenvironment (TME), contributing to the plasticity and heterogeneity of the tumor 1. Cellular heterogeneity has been implicated in reducing therapeutic responses to anti-cancer drugs by conferring fitness advantages to cancer cells 2-6. Thus, understanding the heterogeneity of tumor immune environments and the molecular mechanisms influencing the TME is crucial for the development of novel therapeutic strategies with consistent patient responses.

Among the cellular components of the TME, tumor-associated-macrophages (TAMs) play a crucial role in primary tumor growth and metastasis, either by directly promoting the invasive capacity of cancer cells 7-9, or by indirectly establishing a protumor microenvironment 10-12. In particular, TAMs are widely known as one of the central myeloid cells that establish immunosuppressive TMEs, along with myeloid-derived suppressor cells (MDSCs) and regulatory T cells (Tregs) 13-15. TAM-mediated immunosuppressive niches impair T cell boosting and contribute to resistance to immune checkpoint inhibitors (ICIs) in cancer patients 16-19. In this regard, TAMs have attracted significant attention as a cellular target to overcome tumor immune evasion and immunotherapy resistance.

Therapeutic strategies that target TAMs have evolved significantly and are mostly aimed at depleting TAMs or reprogramming them toward an antitumoral phenotype 20-22. An example of depleting TAMs is the use of bisphosphonates. One of these bisphosphonates, clodronate exhibits antitumor properties with reduced TAMs tumor infiltration, tumor burden and metastasis in various preclinical tumor models 20, 23, 24. Also, there are targets aimed at depletion, such as colony-stimulating factor 1 (CSF1)/ CSF1 receptor (CSF1R) 13, 25-27, C-C motif chemokine 2 (CCR2) 28-30, CCR5 31-33, and phosphatidylinositol 3-kinase γ (PI3Kγ) 15, 34-36. However, due to the functional diversity of TAMs, which can be either protumor or antitumor effectors 37-40, a number of limitations are associated with the broad depletion of TAMs in terms of the therapeutic strategy 41. Furthermore, reported toxicity, probably due to depletion of tissue-resident population important for homeostasis, precludes the use of these TAMs-targeted agents for a long period of time 21, 42, 43. Alternative attempts at repolarizing TAMs have displayed promising therapeutic outcomes in preclinical studies 14, 15, 44, but the clinical application of these approaches still remains challenging. Redundant immunosuppressive functions of other cells also exist as a compensatory mechanism by which cancer cells maintain the immunosuppressive TME, resulting in reduced antitumoral responses to TAM-directed therapies. Thus, the identification of the most relevant protumor functions of TAMs and the specific molecular mechanisms underlying TAM-mediated tumor-supportive TME might enable improved targeted therapies. However, current approaches have not fully considered TAM-mediated immunosuppression stemming from interconnected cellular components within the TME. Thus, a comprehensive analysis for the landscape of various TME components affected by TAMs is highly desirable.

Herein, we used single-cell RNA sequencing (scRNA-seq) to comprehensively examine the transcriptional changes of cancer cells and immune cells within the early TME of 4T1 mouse breast cancer models in the context of TAM depletion using clodronate-loaded liposomes. scRNA-seq revealed that depleting TAMs attenuated the activation of epithelial-to-mesenchymal transition (EMT) in cancer cells, which reduced metastatic potential. We also identified Galectin-1 (Gal-1) as a previously unrecognized molecule associated with TAM-mediated suppression of antitumor immunity. We suggest targeting Gal-1 as a combinational anti-cancer immunotherapeutic approach due to its role in TAM-mediated immunosuppression in the TME.

## Materials and methods

### Mouse breast cancer model and treatments

Breast carcinoma cell lines 4T1-Luc2 and E0771 were maintained in RPMI 1640 medium supplemented with 10% fetal bovine serum (FBS) and 1% Penicillin/Streptomycin (PS) (Gibco). To establish an orthotopic implantation model, tumor cells (3 × 10^5^ cells) were orthotopically implanted into the fourth mammary fat pad of eight-week-old female BALB/c (4T1-Luc2) or C57BL/6 mice (E0771). Macrophage depletion was performed by intraperitoneal injection of clodronate on days 3, 5 and 7 after 4T1 implantation. Controls were administered vehicle liposomes (Encapsula Nano Science). For OTX008 treatment, 4T1-bearing mice were intraperitoneally treated with 5 mg/kg OTX008 (MedChemExpress, Monmouth Junction, NJ, USA) and/or 100 μg anti-PD-1 (RMP1-14; Bio X Cell) five times at 3-day intervals. Corresponding 100 μg isotype antibodies (Bio X Cell) were used for vehicle controls. Tumor burden was evaluated through digital caliper measurement every 2-3 days following the start of treatment and calculated as tumor volume using the following formula: length x width^2^ /2. Lung metastasis was assessed by bioluminescence signals using the IVIS Spectrum *In vivo* Imaging System (PerkinElmer).

All animal experiments were conducted in accordance with the Institute for Experimental Animals College of Medicine and were cared for according to the Guide for the Care and Use of Laboratory Animals prepared by the Institutional Animal Care and Use Committee of Seoul National University (accession number SNU-211003-1).

### Immunofluorescence

Mouse mammary tumor tissues were embedded in optimal cutting temperature (OCT) compound, frozen and stored at -80 °C. Frozen tissue samples were fixed in 4% paraformaldehyde for 15 min and washed with phosphate buffered saline (PBS), then blocked with PBS containing 3% bovine serum albumin (BSA) and 0.3% Triton X-100 (Sigma-Aldrich) for 1 h at room temperature. Tissue sections were then rinsed in staining buffer (1% BSA and 0.3% Triton X-100 in PBS) and incubated overnight at 4 °C with primary antibodies (anti-Gal-1 (Abcam) and anti-CD8 (Thermo Fisher Scientific)) diluted in PBS containing 1% BSA and 0.3% Triton X-100. After washing with PBS thrice, the tissue sections were stained with the appropriate secondary antibodies and 4,6-diamidino-2-phenylindole (DAPI; Invitrogen) for 1 h. Staining controls were stained with secondary fluorescently labelled antibodies and DAPI without primary antibodies. To detect apoptotic cells in tumor sections, cryopreserved sections were stained with an In Situ Cell Death Detection Kit (Roche) according to the manufacturer's protocol. Fluorescent images were acquired on a Leica TCS SP8 confocal microscope (Wetzlar, Germany).

### Quantitative real-time polymerase chain reaction

Total RNA was extracted using TRIzol reagent (Invitrogen) according to the manufacturer's instructions. cDNA was synthesized from 1 μg of total RNA using reverse transcription, and the amount of mRNA was quantified using real-time PCR analysis with SYBR Green qPCR Pre-Mix (Enzynomics, Daejeon, South Korea) on an ABI real-time PCR 7500 machine (Applied Biosystems, CA, USA). Gene expression was normalized to housekeeping gene *18sRNA*. Primer sequences are listed in Table S2.

### Protein extraction from tumor samples

Snap‐frozen tissue samples were cutted into small pieces and homogenized with TissueLyser II and stainless-steel beads (Qiagen GmBH, Hilden, Germany) in RIPA buffer supplemented with Xpert phosphatase inhibitor cocktail solution (GenDEPOT, Barker, TX, USA) and Xpert protease inhibitor cocktail solution (GenDEPOT, Barker, TX, USA) on ice. Following incubation and mixing at 4°C for 30 min, the extract was centrifuged at 12,000 g for 30 min at 4°C. Protein concentrations were determined using Pierce BCA Protein Assay Kit (Thermo Fisher Scientific, Waltham, MA, USA) using supernatant. The supernatant was stored in aliquots at -80°C.

### Western blot analysis

Loading sample buffers were constituted with 15ug (Gal-1 and vimentin) or 30ug (E-cadherin and IGFBP5) protein, LDS sample buffer and sample reducing agent (Novex, San Diego, CA) and boiled 70°C for 10 min. Sample buffers containing 30 ug protein were separated on 4-12% Bis-Tris plus gels (Invitrogen, Carlsbad, CA, USA) and transferred into PVDF membranes using power blotter select transfer stacks (Invitrogen, Carlsbad, CA, USA). The membranes were blocked in blocking buffer (PBS with 5% skim milk, 0.05% Tween 20) for 1 hr at room temperature, followed by incubation with primary antibodies overnight at 4°C. The following primary antibodies were used: anti-Gal 1 (Abcam), anti-vimentin (Cell signaling), anti-E-cadherin (Cell signaling) and anti-IGFBP5 (Santa Cruz). The following day, the membranes were incubated with HRP-conjugated secondary antibodies. Chemiluminescence was detected with an Amersham Imager 680 instrument (GE Healthcare, Piscataway, NJ, USA).

### Enzyme-linked immunosorbent assay (ELISA)

Secreted Gal-1 protein levels were measured in serum samples from tumor-bearing mice using a DuoSet ELISA kit (R&D Systems) according to the manufacturer's protocol.

### Bone marrow-derived macrophage

Bone marrow cells were obtained from the femur and tibia of 7-12-week-old C57BL/6 mice and differentiated into bone marrow-derived macrophages (BMDMs) for 7 d in RPMI 1640 medium containing 10 % FBS, 100 U/mL penicillin, 100 μg/mL streptomycin (Invitrogen, CA USA), and 2 mM of L-glutamine (Invitrogen), and supplemented with fresh recombinant murine colony-stimulating factor 1 (CSF1; 50 ng/mL; Miltenyi Biotec, CA, USA). The medium was changed on days 3 and 5 with fresh medium containing CSF1.

### T cell proliferation assay

Splenocytes isolated from fresh mouse spleen tissue were labeled with the CellTrace Violet dye (Thermo Fisher Scientific) according to the manufacturer's instructions. The labeled cells were seeded in 96-well round-bottom plates at 5 x 10^5^ cells/mL and then incubated for 96 h with TES from ctrl- and clodronate-treated tumors supplemented with Dynabeads mouse T-activator CD3/CD28 (Thermo Fisher Scientific). For co-culture, labeled cells were co-cultured with BMDMs (1:4 ratio of BMDMs to T cells) in RPMI 1640 medium supplemented with 10% FBS and Dynabeads mouse T-activator CD3/CD28. After 96 h at 37 °C, cells were stained with CD4 PE (GK1.5; eBioscience) and CD8a APC-Cy7 (53.6.7; BioLegend) for 30 min at 4 °C. T cell proliferation was determined by CellTrace Violet dye dilution with flow cytometry analysis.

### Flow cytometry

Mammary tumor tissue samples were harvested and mechanically dissociated to generate single-cell suspensions. Thereafter, erythrocytes were removed, and the resulting single cell suspensions were incubated with purified anti-CD16/CD32 (BioLegend) for 15 min at 4 °C, and finally processed for cell-surface staining with the appropriate antibodies at 4 °C. For intracellular staining, cells were fixed using 1% paraformaldehyde (Merck, NJ, USA) and permeabilized using Perm/Wash buffer (BD Biosciences), and finally labeled with the appropriate secondary antibodies. The following antibodies were used: Gal-1 (polyclonal) from Abcam (Cambridge, UK); CD45 FITC (30-F11), CD45 APC (30-F11), CD11b APC (M1/70), Gr 1 V450 (RB6-8C5), Ly6C PerCP-Cy5.5 (HK1.4), F4/80 PE (BM8), CD3 FITC (145-2C11), CD4 PE (GK1.5), Foxp3 V450 (FJK-16s), and Granzyme B V456 (NGZB), CD140a PE (APA5) from eBioscience; EpCAM FITC (G8.8), CD11c BV605 (N418), CD3 PE-Cy7 (145-2C11), CD8a APC-Cy7 (53.6.7), CD19 FITC (6D5), CD4 BV650 (GK1.5) and IFNγ PerCP-Cy5.5 (XMG1.2) from BioLegend; CD31 APC (MEC13.3) and CD8a BV711 (53.6.7) from BD Biosciences. For Annexin staining, cells were washed and stained using the PE Annexin V Apoptosis Detection Kit (BD Biosciences) according to the manufacturer's protocol. Data were acquired using the LSR Fortessa system (BD Biosciences) and analyzed with FlowJo software (Tree Star, OR, USA).

### Fluorescence-activated cell sorting

To sort the intratumoral CD45^+^ EpCAM^-^ cells and CD45^-^EpCAM^+^ cells (cancer cells), single cell suspensions of digested tumors were blocked with purified anti-CD16/CD32 for 10 min at 4 °C and stained for 30 min at 4 °C with the following antibodies: CD45 APC (30-F11; eBioscience), and EpCAM FITC (G8.8; eBioscience). Next, cells were washed and incubated with DAPI to stain dead cells. Sorting of live CD45^+^EpCAM^-^ cells and CD45^-^EpCAM^+^ cells was performed on a BD AriaIII (BD Biosciences).

### Single-cell RNA sequencing

Fluorescence-activated cell sorting (FACS)-sorted cells were loaded onto a 10x Chromium microfluidics system according to the manufacturer's guidelines. As per the guidelines, scRNA-seq 5′ gene expression (GEX) libraries were generated using the 10x Genomics Chromium Single Cell 5′ Kit version 3.1 and the 10x Chromium Controller (10x Genomics) according to the 10x Single Cell 5′ version 3.1 protocol guidelines (CG000204). Briefly, cell suspensions were diluted in nuclease-free water to achieve a targeted cell count of 10,000. Cell suspensions were mixed with master mix and loaded with Single Cell 3'V3.1 Gel Beads and Partitioning Oil into a chromium Next GEM Chip G. RNA transcripts from single cells were uniquely barcoded and reverse-transcribed within droplets. The products were purified and enriched using PCR to create the final cDNA library. The purified libraries were quantified using qPCR and qualified using the Agilent Technologies 4200 TapeStation. The libraries were sequenced using a HiSeq platform (Illumina) according to the read length specified in the user guide. The Cell Ranger v.3.1.0 was used to perform alignment, filtering, barcode and UMI counting with default parameters.

### Analysis of scRNA-seq data

After the estimation of gene counts, data were loaded using Seurat (version 3.1.2). Two datasets of scRNA-seq were merged using canonical correlation analysis (CCA) from the Seurat package. The cells were filtered for further analysis based on the following parameters: expression of at least 200 genes and 6000 genes at most to exclude cell duplets, and mitochondrial genes accounting for less than 15% of the transcripts. The transcript counts were log transformed with multiplication of scaling factor 10,000. Variable features (nfeatures = 2000) were identified based on a variance stabilizing transformation. Principal component analysis (PCA) was run on variable genes, and 50 PCs were selected for clustering analyses. Cells were clustered using the FindClusters function in Seurat with default settings and a resolution of 0.6. The marker genes of each cluster were identified by the FindAllMarkers function in Seurat. For data visualization and dimension reduction, uniform manifold approximation and projection (UMAP) embedding was used.

The cell types of clusters were defined according to known marker genes. Major groups of cell types were defined as: B cells, cancer cells, CD4 T cells, CD8 T cells, other T cells, myeloid cells, natural killer (NK) cells, and dendritic cells. To define the cell subclusters of each major cell type, particularly myeloid cells, scType was used 45.

To identify differentially expressed genes (DEGs) between the clodronate and control groups, MAST implemented in the Seurat package was used 46. After the extraction of DEGs, Gene ontology (GO) analysis was performed using the cluster Profiler package. The input genes for GO were selected by FDR-corrected *p* < 0.05.

The trajectory analysis for cancer cell subclusters was performed using Monocle 3 47. Using the cancer cell subset, a graph trajectory was estimated through UMAP space. The beginning of pseudotime was selected as the branch node of an enriched cluster in the control. The trajectory and pseudotime were estimated for cancer cells.

To estimate cell-to-cell interactions, particularly prioritizing ligands for the DEGs of cancer cells in the clodronate group, we utilized NicheNet 48. DEGs were filtered using the following parameters: *p* < 0.01, and absolute log FC > 0.25. From the receptor-ligand interaction database, ligands and receptors were selected when receiver and sender cells constituted at least 10% of the cells. Cancer cells were selected as receiver cells and other cell types were selected as sender cells. Prioritized ligands were estimated using the 'predict_ligand_activities' function of NicheNet.

### scRNA-seq of human breast cancer during anti-PD-1

A single cell map of intratumoral changes during anti-PD-1 usage in human breast cancer was used 49. Cell clustering and UMAP embedding for visualization were performed using Seurat. Cell annotations that were provided by authors were used for further analyses. Triple negative breast cancer types were selected and Gal-1 expression levels were evaluated for the clusters.

### Statistics

All statistical analyses were performed using GraphPad Prism v.9.3. Data were presented as mean ± standard error of the mean (SEM), and *p* < 0.05 was considered statistically significant. Two-tailed unpaired student's *t*-tests or two-way analysis of variance (ANOVA) tests were used to determine statistical significance. All experiments were performed at least twice, with similar results obtained each time. Figure legends denote the specific statistical tests used for each experiment.

## Results

### TAM depletion reorganizes immune cells in TME and suppresses metastatic potential

To explore the changes in cancer cells and immune cells within the TME in response to TAM depletion during the early stages of tumor progression, we administered clodronate in a 4T1 syngeneic orthotopic breast tumor model and examined the population dynamics using flow cytometry at an early time point (after 4^th^ injection of clodronate on day 13). At this time point, a slight decrease in the tumor burden was observed (Figure S1A). There was a dramatic decrease in cancer cells in clodronate-treated mice when compared to vehicle-treated controls; however, the percentage of CD45^+^ cells was not significantly changed (Figure S1B). Clodronate treatment resulted in a decrease in CD11b^+^ myeloid cells, specifically a marked reduction in macrophages (Figures S1C and S1D). Other CD11b^+^ myeloid cell levels, including monocytes and neutrophils, remained unchanged or were elevated (Figures S1E and S1F).

**Figure 1:**
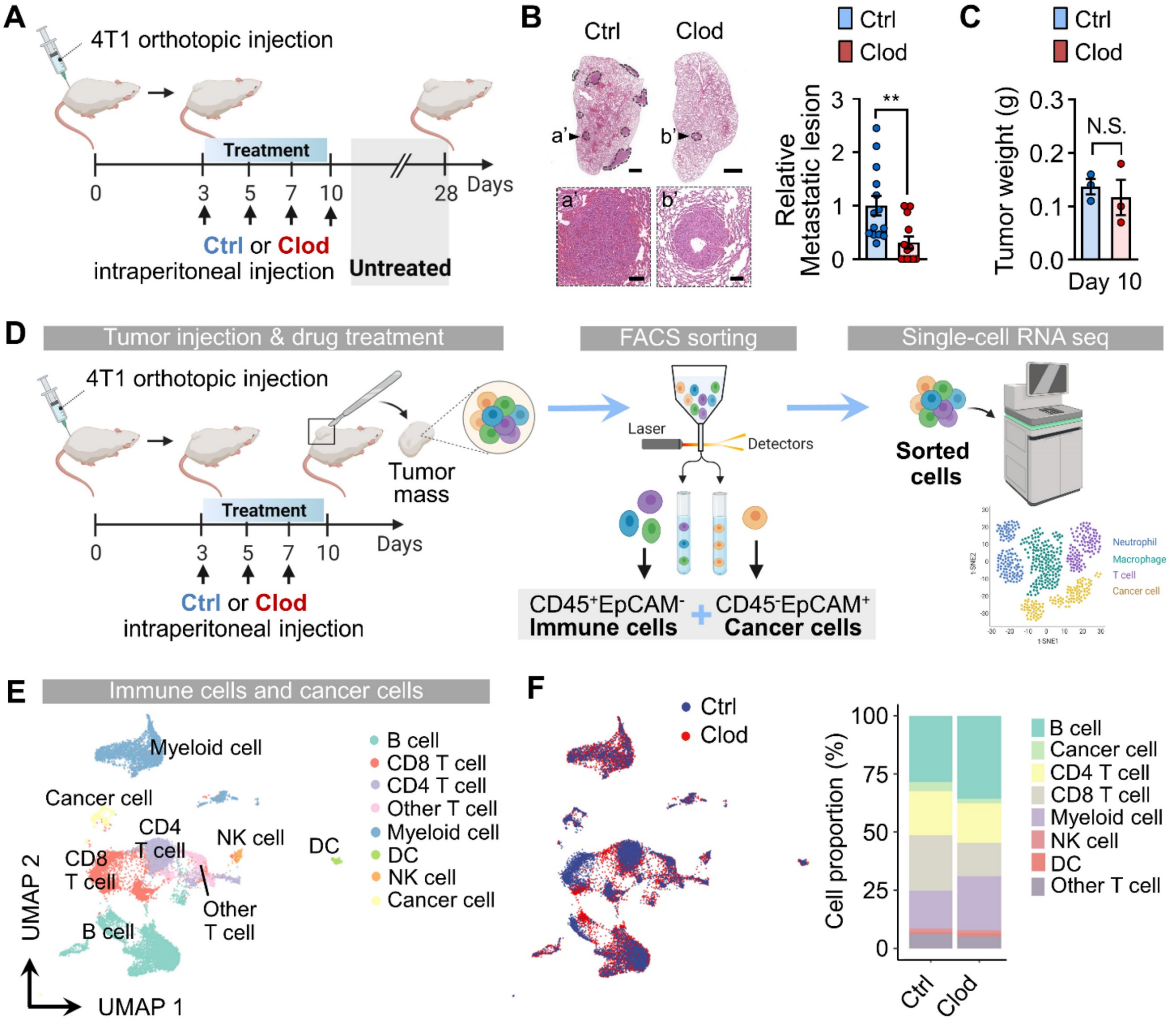
** Tumor-associated macrophages (TAMs) depletion reorganizes immune cells in tumor microenvironments (TMEs) and suppresses metastatic potential.** (A) Schematic illustration of analysis in 4T1 tumor-bearing mice treated with either clodronate (clod) or vehicle for 10 days; treatment was then halted until day 28. (B) Representative hematoxylin and eosin (H&E)-stained lung images (left) and quantification of metastatic lesion area in the lungs (right) of 4T1 tumor-bearing mice, as described in Figure 1A. The dotted lines indicate metastatic nodules in the lung. Scale bar, 1 mm; inset scale bar, 100 μm. (C) Tumor weight in 4T1-tumor bearing mice after vehicle (control, n = 3 mice) or clodronate (clod, n = 3 mice) treatment every 2-3 days for 7 days. (D) Schematic illustration of analysis of scRNA-seq data. (E) Uniform manifold approximation and projection (UMAP) of total cells, including immune cells and cancer cells, colored to display the annotated cell types. (F) UMAP plots of identified cell types, colored based on treatment with clod or vehicle (left). Cell proportion of the eight major cell types were represented (right). All data represented as mean ± S.E.M. Statistical significance was determined by two-tailed *t*-test. **P < 0.01. N.S., nonsignificant.

Next, we aimed to determine the impact of TAM depletion on metastasis formation during the early tumor stages. To this end, we depleted TAMs via clodronate treatment for 10 days and then left the mice untreated until day 28, which is when tumor cells spontaneously metastasized in the lungs (Figure 1A). We then compared the metastatic lesions in the lungs from tumor-bearing mice that received clodronate to those in control mice. Although overt tumor growth was not affected by clodronate treatment by the end of the experiment (day 28) (Figure S1G), transient depletion of TAMs during the early stage was sufficient in reducing metastasis formation in the lungs (Figure 1B). These findings suggested a causal role of TAMs in endowing early cancer cells with metastasis-initiating capabilities.

To elucidate the mechanisms of TAM-mediated early cellular repopulation and transcriptional programs that precede detectable changes in tumor growth, we performed scRNA-seq using samples from tumors at day 10 after 4T1 implantation. At this time point, no significant difference in tumor weight was observed (Figure 1C), despite a modest decrease in macrophage accumulation (Figure S1H). First, we collected cancer cells (CD45^-^EpCAM^+^) and immune cells (CD45^+^EpCAM^-^) via FACS from the tumor mass of 4T1 tumor-bearing mice either with or without clodronate treatment (Figure S2). The sorted cells were subjected to scRNA-seq using the 10x Genomics platform (Figure 1D). By analyzing a total of 16,491 cells (8,099 cells for the control group and 8,392 cells for the clodronate group) with the UMAP algorithm, we identified 22 different clusters (Figures S3A-S3C), which were assigned to eight major cell types expressing representative known marker genes (Figure 1E). The proportional changes of annotated cell types differed between the two groups (Figures 1F and S3D).

### TAM depletion leads to decreased EMT signature in cancer cells

**Figure 2:**
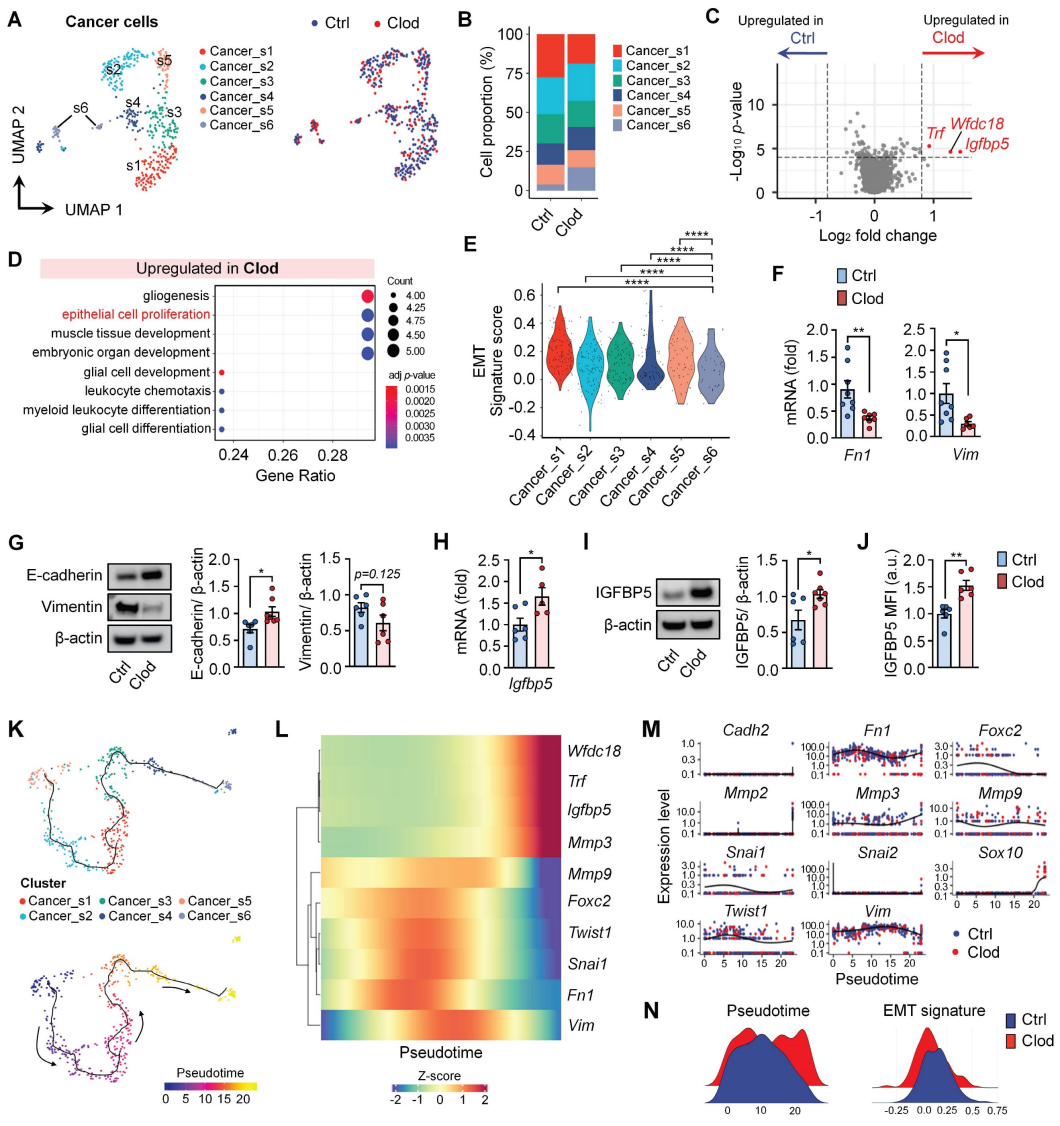
** TAM depletion leads to a decreased epithelial-to-mesenchymal transition (EMT) signature in cancer cells.** (A) UMAP plots of cancer cells, colored to identify each subcluster (left) and treatment with clodronate (clod) or vehicle (ctrl) (right). (B) Proportion of each subcluster of cancer cells between two experimental groups. (C) Volcano plot showing differentially expressed genes (DEGs) in cancer cells from tumors treated with either clod or vehicle. (D) Enriched gene ontology (GO) functions of upregulated genes in cancer cells from clod group. (E) Violin plots showing signature scores for EMT in each cancer cell subcluster. Statistical significance between different clusters was determined by a non-parametric Mann-Whitney U test. (F) mRNA expression of mesenchymal feature-related genes, fibronectin (*Fn1*), and vimentin (*Vim*) in tumor tissues from 4T1-bearing mice treated with clod (n = 6-7 mice) or vehicle (n = 8-9 mice). (G) Western blot analysis (left) and quantification (right) of E-cadherin and vimentin in tumor lysate upon treatment with clod or vehicle. (H) *Igfbp5* mRNA expression in tumor tissues from 4T1-bearing mice treated with clod (n = 5 mice) or vehicle (n = 6 mice). (I) Western blot analysis (left) and quantification (right) of IGFBP5 in tumor lysate. (J) Flow cytometric analysis of the expression of IGFBP5 in cancer cells, expressed as the mean fluorescence intensity (MFI) (n = 6~7 mice per group). (K) Pseudotime trajectory analysis was performed on scRNA-seq data of cancer cells. According to the trajectory, pseudotime was defined according to the direction to the Cancer_s6 cluster. (L and M) Genes involved in EMT signatures and DEGs were represented according to the pseudotime. (N) The scores of the pseudotime and the EMT signature were plotted for the two experimental groups. All data represented as mean ± S.E.M. Statistical significance was determined by two-tailed *t*-test. *P < 0.05, **P < 0.01 and ****P < 0.0001.

Subclusters of cancer cells were investigated to examine the changes in gene expression after clodronate treatment. Among EpCAM^+^ cancer cells, six subclusters were identified from the UMAP (Figures 2A and S4A). We found that TAM depletion led to an increase in the proportion of the Cancer_s6 cluster, which expressed higher levels of genes *Igfbp5, Wfdc18, S100a9, Il1b,* and *S100a8* (Figures 2B and S4A). Furthermore, the DEGs of cancer cells from clodronate-treated tumors (*Wfdc18*, *Igfbp5,* and *Trf)* corresponded to the marker genes of the Cancer_s6 cluster (Figure 2C and Table S1). GO term analysis revealed that these cancer cells exhibited an upregulation of pathways associated with epithelial cell proliferation and myeloid leukocyte differentiation following clodronate treatment (Figure 2D). Among them, we focused on upregulated epithelial cell proliferation because it is involved in regulating EMT, which is closely related to the metastatic potential of cancer cells. The mesenchymal gene sets (*Vim, Cdh2, Foxc2, Mmp9, Snai1, Snai2, Twist1, Sox10, Fn1, Mmp2,* and *Mmp3*) were selected to evaluate EMT potential 50. We observed that the Cancer_s6 cluster displayed the lowest transcriptional EMT signature compared to other subclusters (Figure 2E) and the clodronate group exhibited a lower expression of mesenchymal feature-related genes than the control group (Figures S4B and S4C). Consistent with these findings, RT-PCR confirmed the decreased mRNA levels of mesenchymal feature-related genes, including *Fn1* and *Vim,* in tumor tissues after clodronate treatment (Figure 2F). EMT phenotype was further explored by protein detection of E-cadherin and vimentin, two critical markers of the epithelial and mesenchymal status in cancer cells and similarly reversed EMT signature was observed in two different mouse breast cancer models following clodronate treatment (Figures 2G and S5).

We also experimentally validated one of the most significantly upregulated genes, *Igfbp5* using quantitative real-time PCR (Figure 2H), western blotting (Figure 2I) and flow cytometry (Figure 2J). Finally, to investigate the fate of cancer cells upon TAM depletion, pseudotime analysis was applied to scRNA-seq (Figure 2K). Genes identified through differential gene expression analysis and EMT signatures to be overexpressed in cancer cells were plotted (Figures 2L and 2M). We found that pseudotime was relatively increased in the clodronate group compared with the controls (Figure 2N). In the trajectory analysis, the clodronate group exhibited changes in cellular status that moved toward a decreased EMT signature.

### TAM depletion reconstitutes immune cells in TME

**Figure 3:**
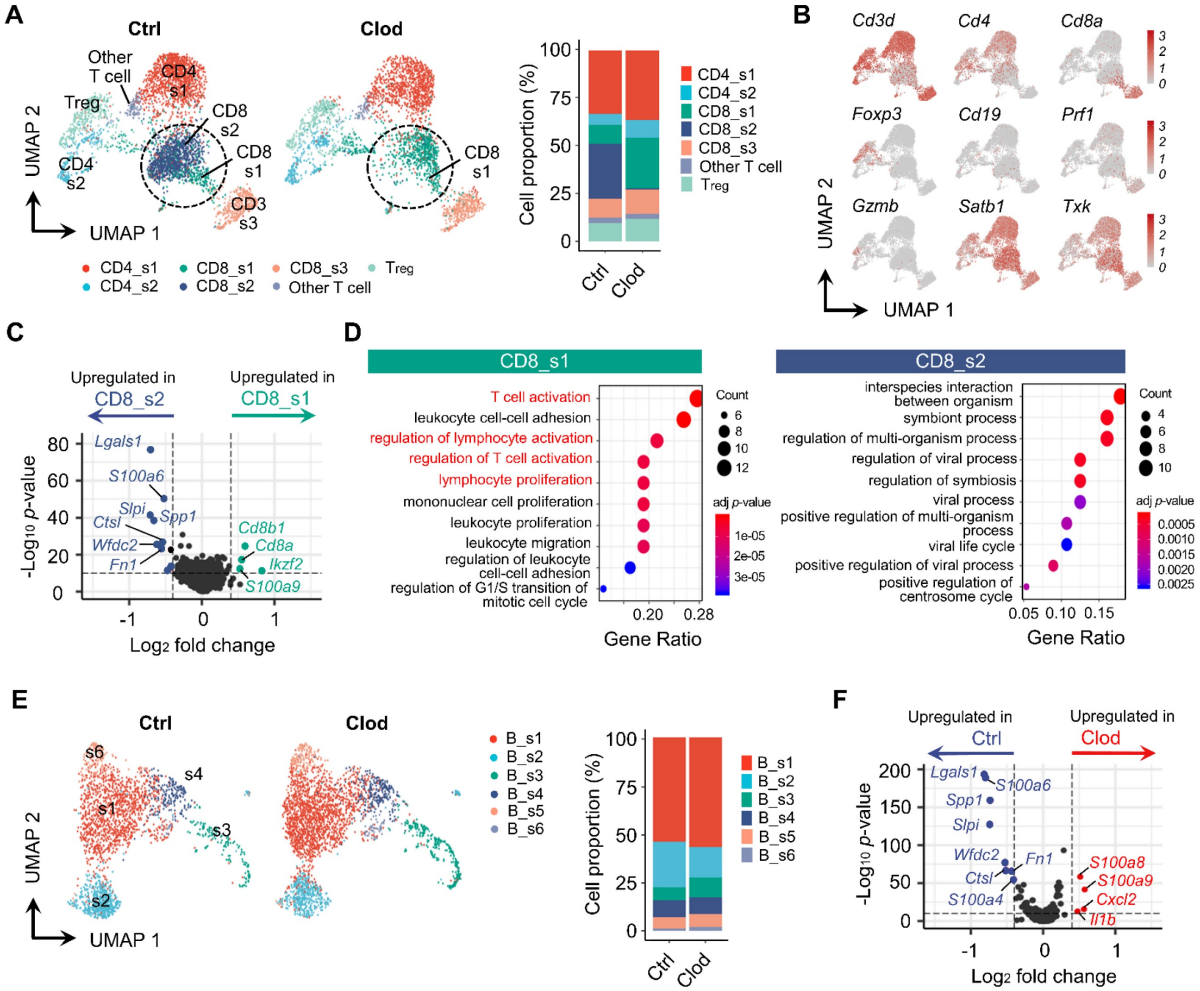
** Reorganized subpopulation of T and B cells after TAM depletion.** (A) UMAP plots of T cells, colored based on subcluster (left) and proportion of each subcluster (right) in tumor following treatment with vehicle (ctrl) or clodronate (clod). (B) UMAP plots of T cells, colored based on the expression of selected cell subtype marker genes. (C) Volcano plot displaying DEGs between CD8_s1 and CD8_s2. *Lgals1* was the top DEG that was highly expressed in CD8_s2. (D) Enriched GO terms of CD8_s1 (left) and CD8_s2 (right). (E) UMAP plots of B cells, identified by each subcluster (left) and proportion of each subcluster (right) in tumors treated with either clod or vehicle. (F) Volcano plot displaying DEGs in B cells from tumors treated with either clod or vehicle.

It has been known that TAMs establish an immunosuppressive TME as a pivotal regulator of T cells 51. Indeed, our data revealed that population of T cells was markedly reorganized in the clodronate group (Figures 3A). The subclusters of T cells were identified from UMAP (Figures 3B and S6A). After comparing the relative proportion of each cluster, distinctive proportional changes were observed in CD8^+^ T cell subtypes. The CD8_s2 cluster was dominant in the control group but was drastically decreased in the clodronate group. In contrast, the clodronate group exhibited an increased proportion of the CD8_s1 cluster. These two subtypes were marked by a high expression of *Satb1* and *Txk* and relatively low expression of *Cd3d* compared with the other T cell subclusters (Figure 3B). Due to the significant decrease of the CD8_s2 cluster in the *Satb1^+^* CD8^+^ T cell population after TAM depletion, we analyzed the DEGs between these two subclusters and found that *Lgals1* encoding Gal-1 was highly expressed in the CD8_s2 cluster, which was also strongly enriched in the control group (Figure 3C). Furthermore, the CD8_s1 cluster was closely related to T cell activation and the regulation of lymphocyte activation, while the CD8_s2 cluster mediated biological functions including interspecies interactions between organisms (Figure 3D). Next, the subclusters of B cells were analyzed (Figures 3E and S6B). Unlike T cells, the changes in proportions of B cells were not shown by TAM depletion when compared to those in the control group (Figure 3E). According to differential gene expression analysis, the control group exhibited upregulation in expression of genes, including *Lgals1*, *S100a6*, *Spp1,* and *Slpi*, which were similarly upregulated in T cells (Figure 3F). GO analyses of the enriched DEGs in B cells are summarized in Figures S6C and S6D.

**Figure 4:**
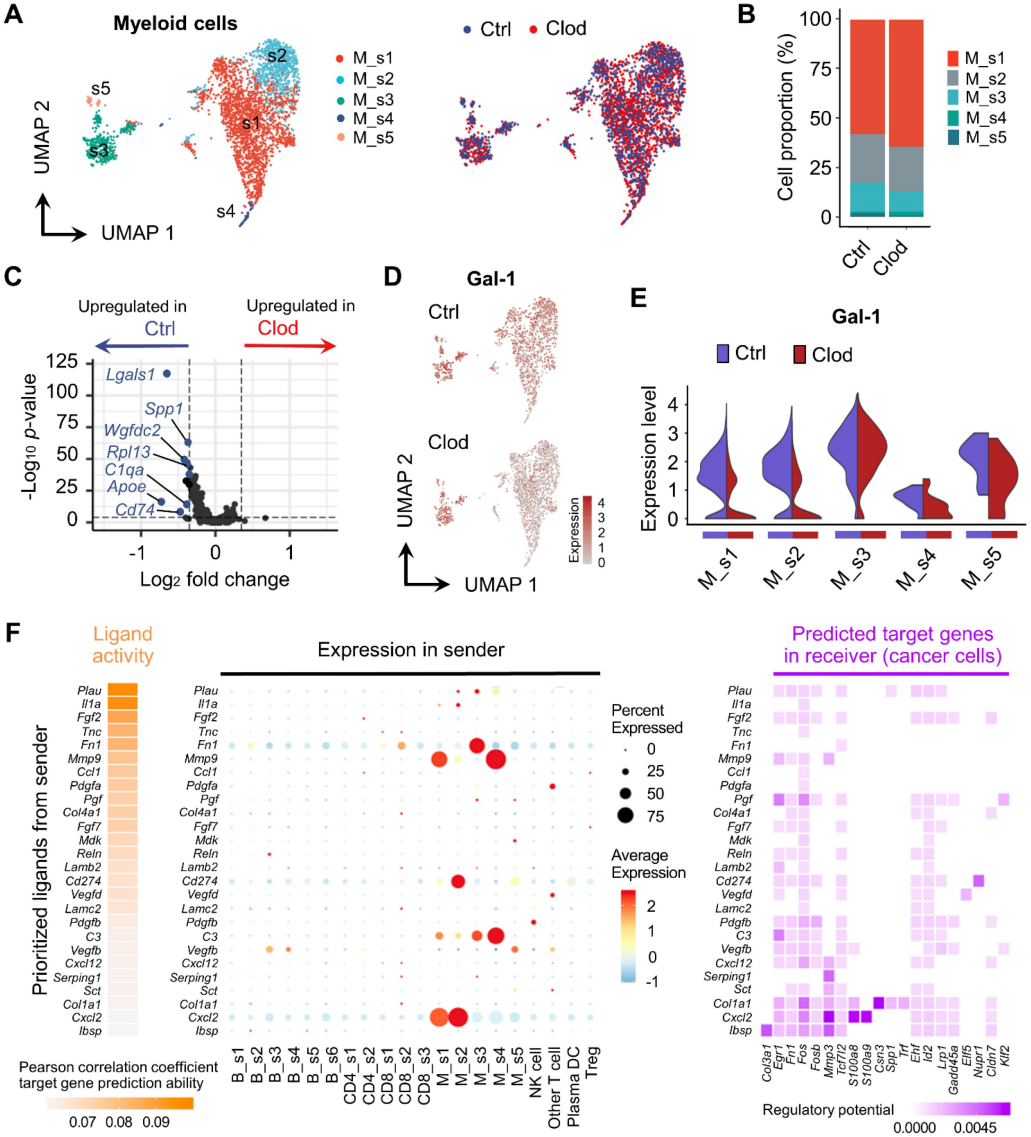
** Molecular changes in myeloid cells after TAM depletion.** (A) UMAP plot of myeloid cells, colored based on subcluster (left) and treatment with clodronate (clod) or vehicle (ctrl) (right). (B) Proportion of each subtype in tumors treated with clod or vehicle. (C) Volcano plot showing DEGs in myeloid cells between the two experimental groups. (D) UMAP plots of Gal-1 expression in myeloid cells. (E) Violin plots comparing the expression distributions of Gal-1 among myeloid cell subclusters from vehicle- versus clod-treated tumors. (F) Ligand activities and predicted target genes for cancer cells as receiver cells, as identified by NicheNet.

We also analyzed the expression differences of myeloid cells upon TAM depletion. We annotated the myeloid cell types based on key markers and scType 45 and identified five different subclusters (Figures S7 and 4A). Although none of the cell type proportions were significantly altered after clodronate treatment (Figure 4B), it significantly changed expression profiles in myeloid cells (Figure 4C). Particularly, a marked reduction in Gal-1 expression was observed in the M_s1 (monocytes) and M_s2 clusters (MDSC), with higher expression levels of *Nos2* and *Cd274* (PD-L1), which are immunosuppression markers (Figures 4D, 4E and S7).

Due to our findings indicating that clodronate treatment affects EMT and the metastatic potential of cancer cells (Figures 2E-2G), we performed NicheNet analysis 48 to predict the putative interactions between cancer cells ("receiver”) and other cellular components ("sender”) within the TME. We found that prioritized ligands with a high impact on cancer gene expression, including *Fn1, Mmp9,* and *Cd274*, were mainly expressed in myeloid cells, suggesting that the molecular changes of cancer cells may be induced by myeloid cells (Figure 4F). In addition, we found that these ligands had the regulatory potential to express *Egr1, Fn1,* and* Fos* in cancer cells, thereby regulating EMT signaling pathways 52, 53. To extend this ligand-receptor analysis to Gal-1, we used a human receptor-ligand database 54 and *PTPRC* and *ITGB1* were identified as the most probable receptors involved in the interaction with Gal-1 (Figure S8A). When we analyzed expression of these receptors in our scRNA-seq dataset, the *Ptprc* expression was the highest in CD8 T cells, while *Itgb1* was predominantly expressed in cancer cells and NK cells (Figure S8B). In interpreting the interaction of Gal-1 with *PTPRC* and *ITGB1*, it is important to consider that Gal-1 can bind a broad array of molecules presenting accessible polyLacNAc ligands on various glycoconjugates, with the binding specificity being highly influenced by the glycosylation patterns which are context-dependent and variable across different cell types 55.

### Gal-1 contributes to TAM-mediated immunosuppressive microenvironments in breast cancer

**Figure 5:**
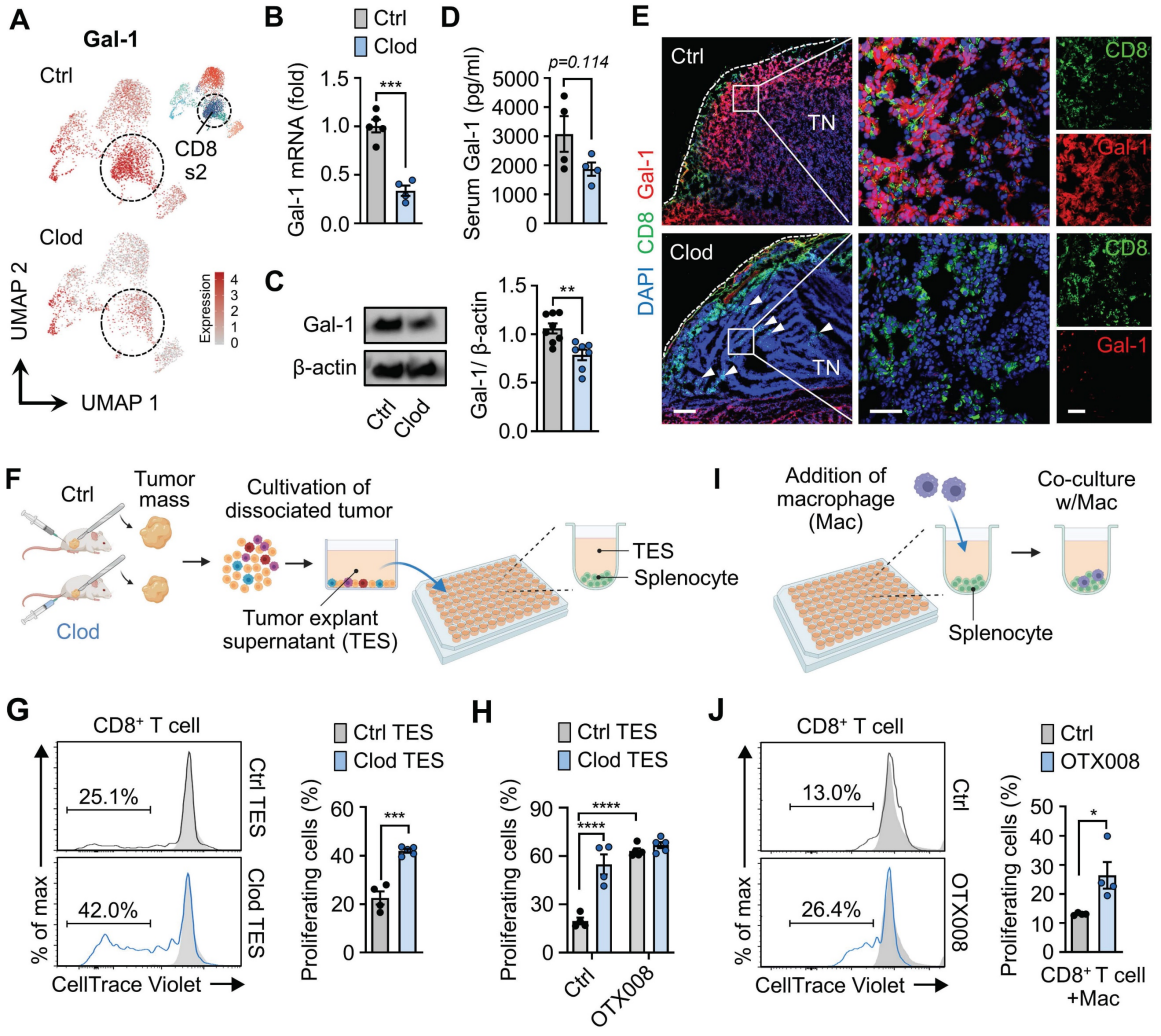
** Gal-1 establishes the TAM-mediated immunosuppressive microenvironment.** (A) UMAP plots of Gal-1 expression in T cells from tumors treated with either clodronate (clod) or vehicle (ctrl). (B) Gal-1 mRNA expression in tumor tissues from 4T1-bearing mice treated with clod or vehicle (n = 4~5 mice per group). (C) Western blot analysis (left) and quantification (right) of Gal-1 in tumor lysate upon treatment with clod (n = 7) or vehicle (n = 8). (D) ELISA for Gal-1 in serum from 4T1-bearing mice (n = 4 mice per group). (E) Representative immunofluorescence staining of CD8 (green) and Gal-1 (red) in the tumor tissues from 4T1-bearing mice treated with clod or vehicle. White dashed lines indicate tumor boundaries; white arrows point to intratumoral CD8^+^ T cell infiltration. Scale bar, 200 μm (left); 50 μm (middle); 50 μm (right). Images are representative of two independent experiments. (F) Schematic illustration of the experimental design for the T cell proliferation assay upon treatment with tumor explant supernatant (TES). (G) Proliferation of activated CD8^+^ T cells exposed to TES in ctrl- (Ctrl TES) or clodronate-treated tumors (Clod TES) (n = 4~5 per group). (H) Proliferation of activated CD8^+^ T cells exposed to Ctrl TES or Clod TES in the presence of vehicle (ctrl) or OTX008 (10μM) (n = 4~5 mice per group). (I) Schematic illustration of the experimental design for the T cell proliferation assay after co-culture with macrophages at a ratio of 4:1. (J) Proliferation of activated CD8^+^ T cells in co-culture with macrophages in the presence of vehicle or OTX008 (10μM) (n = 4 per group). All data represented as mean ± S.E.M. Statistical significance was determined by two-tailed *t*-tests for B-D, G, and J and two-way ANOVA for H. *P < 0.05, ***P < 0.001, and ****P < 0.0001. TN, tumor nest.

Our analyses on each immune cell type revealed that an immunosuppressive molecule Gal-1 is significantly under-transcribed in clodronate-treated tumors. Notably, we also found that the CD8_s2 cluster, which was dramatically reduced upon macrophage depletion, exhibited increased Gal-1 expression (Figure 5A). To validate Gal-1 expression according to clodronate treatment, RT-PCR and western blot were performed. We observed significant downregulation of Ga1-1 mRNA and protein levels in tumor tissues upon clodronate treatment (Figures 5B and 5C). Moreover, the depletion of TAMs resulted in reduced Gal-1 secretion in serum, indicating alterations in the systemic production and secretion of Gal-1 by TAMs (Figure 5D). Importantly, this decreased level of Gal-1 following treatment was the most prominent in CD8^+^ T cell, while the other cells showed little or modest changes in Gal-1 expression (Figure S9). Consistent with these data, immunostaining also confirmed that the expression of Gal-1 was significantly higher in vehicle-treated tumors than that in clodronate-treated tumors (Figure 5E). CD8^+^ T cells exhibited high Gal-1 expression, with the expression being reduced by clodronate administration. The depletion of TAMs was followed by an increase in CD8^+^ T cells infiltrating tumor nests.

Based on these results, we investigated whether Gal-1 was functionally involved in TAM-mediated CD8^+^ T cell exhaustion, as revealed by scRNA-seq. First, to confirm the direct effects of macrophage depletion on T cell activation, T cell proliferation assays were performed. We collected the supernatants of tumor explants (TES) from tumors treated with either clodronate (clod) or vehicle (ctrl), and CD8^+^ T cells purified from splenocytes were exposed to TES (Figure 5F). Expectedly, CD8^+^ T cells treated with ctrl-TES exhibited a significant decrease in proliferation compared to those treated with clod-TES, suggesting a possible involvement of macrophages in regulating CD8^+^ T cell proliferation and activation (Figure 5G). Notably, Gal-1 blockade via OTX008 treatment (currently undergoing clinical trial 56) reversed CD8^+^ T cell suppression, resulting in enhanced proliferation at levels comparable to those in the clod group (Figure 5H).

Next, given that TES was derived from the complex system within tumors including cancer cells as a major contributor to Gal-1 in TME, we further assessed the impact of Gal-1 on the immunosuppressive capacity of macrophages by co-culturing macrophages with T cells (Figure 5I). When the co-culture was supplemented with OTX008, the inhibitory effects on CD8^+^ T cell proliferation were reversed (Figures 5J and S10A), whereas no effects on CD8^+^ T cells cultured without macrophages were observed (Figure S10B).

### Pharmacological inhibition of Gal-1 reinvigorates dysfunctional CD8^+^ T cells within tumors

**Figure 6:**
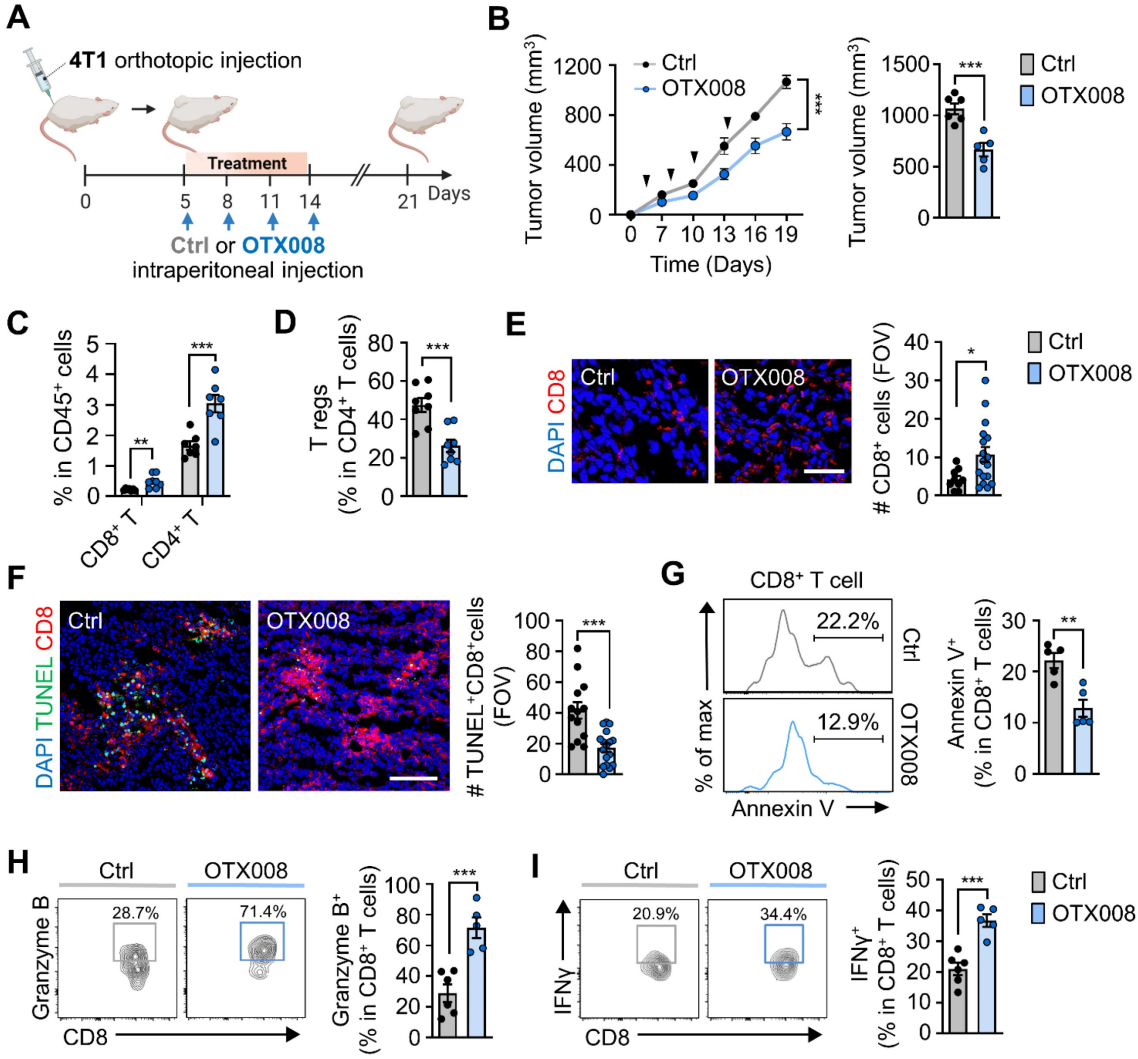
** Pharmacological inhibition of Gal-1 reinvigorates intratumoral dysfunctional CD8^+^ T cells.** (A) Schematic illustration of analysis of tumor growth in 4T1-tumor bearing mice treated with OTX008 (5 mg/kg, n = 5 mice) or vehicle (ctrl, n = 6 mice). (C) Tumor growth of 4T1 cells inoculated orthotopically in syngeneic mice treated as described in A. The follow-ups of tumor size (left) and final tumor volumes at day 21 (right) are shown. (C and D) Flow cytometric quantification of tumor-infiltrating CD8^+^, CD4^+^ T cells (C), and Tregs (D) (n = 7~8 mice per group). Data are expressed as the percentage of CD45^+^ cells, except for Tregs, which are expressed as the percentage of total CD4^+^ T cells. (E) Representative immunofluorescence staining (left) and quantification of CD8 (red) per field of view (FOV) (right) in tumor tissues from 4T1-bearing mice treated with OTX008 or vehicle. Scale bar, 50 μm. (F) Representative immunofluorescence staining (left) of terminal deoxynucleotidyl transferase dUTP nick end labeling (TUNEL) (green) and CD8 (red) and quantification of TUNEL^+^CD8^+^ (right) in the tumor tissues. Scale bar, 100 μm. Images are representative of two independent experiments. (G) Representative flow cytometric analysis of annexin V (left) and frequency of annexin V^+^ (right) in CD8^+^ T cells from tumor-bearing mice treated with OTX008 (n = 5 mice) or vehicle (n = 5 mice). (H) Representative flow plots (left) and percentages of granzyme B^+^CD8^+^ T cells (right) in 4T1-bearing mice treated with OTX008 (n = 5 mice) or vehicle (n = 6 mice). (I) Representative flow plots (left) and percentages of IFNγ^+^CD8^+^ T cells (right) in 4T1-bearing mice treated with OTX008 (n = 5 mice) or vehicle (n = 6 mice). All data represented as mean ± S.E.M. Statistical significance was determined by two-tailed *t*-tests. *P < 0.05, **P < 0.01 and ***P < 0.001.

Next, we examined the effects of OTX008-induced Gal-1 inhibition on the growth of orthotopically injected breast tumors (Figure 6A). We observed reduced 4T1 tumor growth upon OTX008 treatment when compared to vehicle controls (Figure 6B). Along with the tumor-inhibiting effects, we observed changes in immune cell populations after treatment. OTX008 treatment significantly increased tumor-infiltrating CD8^+^ and CD4^+^ T cell populations, whereas the proportion of intratumoral Tregs among the CD4^+^ T cell population were markedly reduced (Figures 6C and 6D). Proportions of intratumoral myeloid cells, including CD11c^+^ cells, monocytes, macrophages, and neutrophils, were not significantly different in OTX008-treated tumors when compared to those in vehicle-treated tumors (Figure S11). Using immunofluorescence staining of tumor tissues, we confirmed a similar increase in tumor-infiltrating CD8^+^ T cells (Figure 6E) and observed fewer terminal deoxynucleotidyl transferase dUTP nick end labeling (TUNEL)-positive intratumoral CD8^+^ T cells upon OTX008 treatment (Figure 6F). In line with this observation, OTX treatment led to a reduction in CD8^+^ T cell apoptosis, as determined by annexin V staining (Figure 6G). Higher *Cxcl9* and *Cxcl10* mRNA expression was also observed in tumors from OTX008-treated mice (Figure S12). These results were in agreement with the previously described role of Gal-1 in anti-apoptosis and recruitment of CD8^+^ T cells 57, 58. Moreover, we observed an approximately three-fold and two-fold increase in the percentage of CD8^+^ T cells expressing granzyme B or interferon-γ (IFNγ), respectively, following OTX008 treatment (Figures 6H and 6I). Collectively, these results indicate that Gal-1 inhibition augments CD8^+^ T cell reinvigoration, thereby eliciting robust antitumor immune responses in the TME.

### Gal-1 inhibition synergizes with PD-1 blockade to suppress breast tumor growth

To examine the potential translational relevance of our findings from scRNA-seq data, we reanalyzed the recently published scRNA-seq data of paired pre- versus on-treatment biopsies from human breast cancer patients treated with anti-PD-1 49 (Figures S13A and S13B). T cell-nonexpanders exhibited relatively higher Gal-1 expression than T cell-expanders in cancer cells, myeloid cells, and T cells following anti-PD-1 treatment (Figures S13C and S13D).

**Figure 7:**
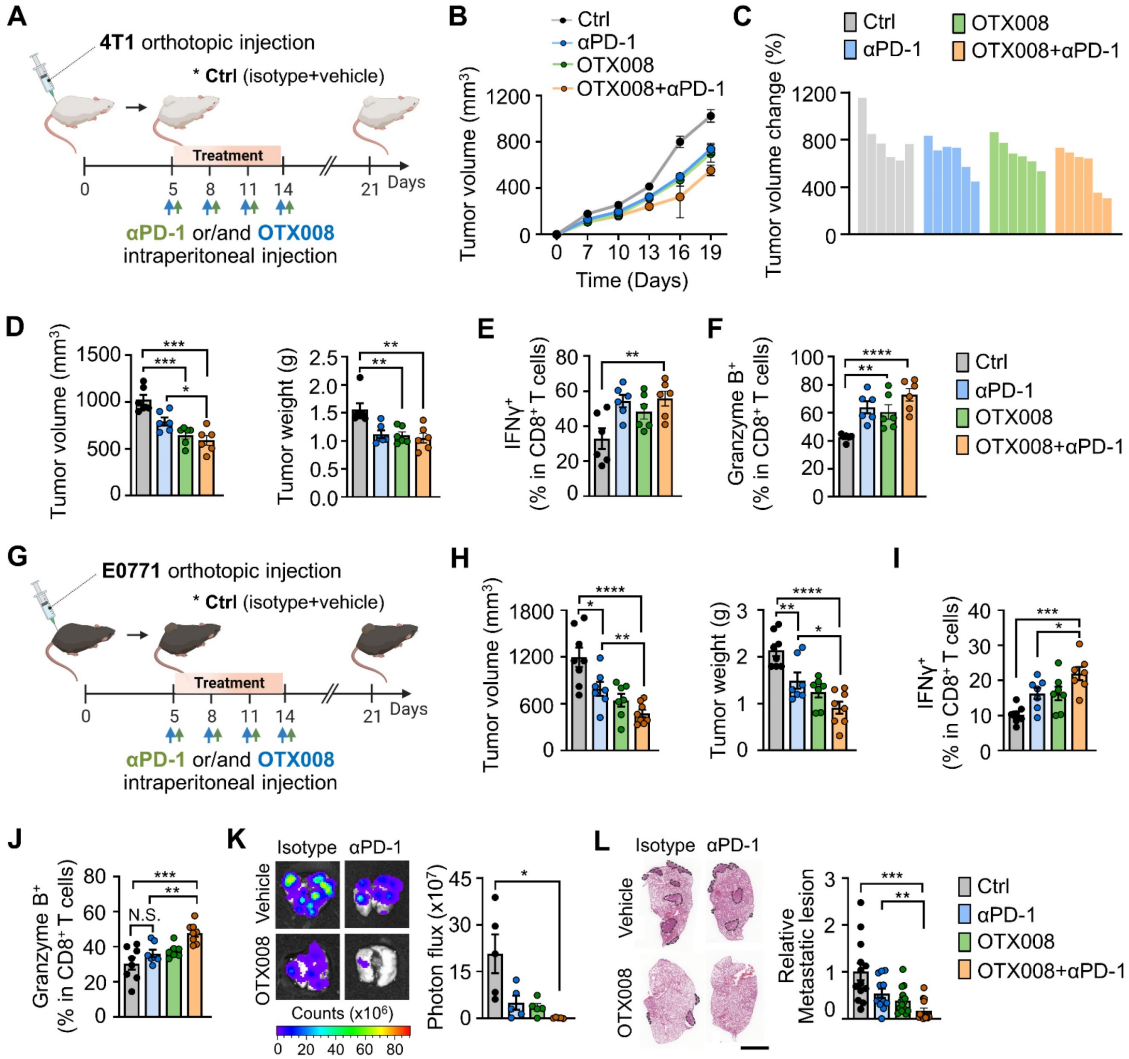
** Gal-1 inhibition sensitizes breast tumors to PD-1 blockade.** (A) Schematic illustration for analysis of tumor growth and lung metastasis in 4T1-tumor bearing mice treated with either anti-PD-1 (100μg, n = 6 mice), OTX008 (5 mg/kg, n = 6 mice), their combination (n = 6 mice), or vehicle (ctrl, n = 6 mice). (B) Tumor growth curves for 4T1-bearing mice treated as indicated. (C) Waterfall plot of individual tumor volume changes (between days 0 and 21). (D) Tumor volume (left) and tumor weight (right) at day 21. (E) Flow cytometric quantification of IFNγ^+^ in tumor-infiltrating CD8^+^ T cells. (F) Flow cytometric quantification of granzyme B^+^ in tumor-infiltrating CD8^+^ T cells. (G) Schematic illustration for analysis of tumor growth in E0771-tumor bearing mice treated with either anti-PD-1 (n = 7 mice), OTX008 (n = 7 mice), their combination (n = 7~8 mice), or vehicle (ctrl, n = 8 mice). (H) Tumor volume (left) and tumor weight (right). (I and J) Flow cytometric quantification of IFNγ^+^ (I) and granzyme B^+^ (J) in tumor-infiltrating CD8^+^ T cells. (K) *Ex vivo* bioluminescence imaging of lungs from 4T1-tumor bearing mice treated with anti-PD-1, OTX008, OTX008+anti-PD-1, or vehicle (n = 5 mice per group). Representative images (left) and quantitative analysis (right) of lung metastasis are shown. (L) Representative H&E-stained lung images (left) and quantification of metastatic lesion areas in the lungs (right) of 4T1-bearing mice as described in K. Dotted lines indicate metastatic nodules in the lung. Scale bar, 3 mm. All data represented as mean ± S.E.M. Statistical significance was determined by two-tailed *t*-tests. *P < 0.05, **P < 0.01, ***P < 0.001 and ****P < 0.0001. N.S., nonsignificant.

Based on the higher expression of Gal-1 in T cell-nonexpanders upon anti-PD-1, we investigated whether Gal-1 inhibition could sensitize tumors to PD-1 blockade therapy in established poor T cell-infiltrated 4T1 breast tumors (Figure 7A). Administration of OTX008 in combination with the PD-1 monoclonal antibody exhibited a stronger antitumor effect compared with vehicle controls and each treatment alone (Figures 7B-7D). The combined OTX008 and anti-PD-1 treatment also led to a significant increase in tumor-infiltrating CD8^+^ (Figure S14A) and CD4^+^ T cells (Figure S14B), whereas intratumoral Treg proportions amongst total CD4^+^ T cells were reduced (Figure S14C). Moreover, the proportion of CD8^+^ T cells expressing IFNγ and granzyme B was significantly increased in the combined treatment group (Figures 7E and 7F). We also investigated the effect of combination treatment in another breast tumor model E0771 (Figure 7G). Similarly, OTX008 synergized with anti-PD-1 to attenuate tumor growth (Figure 7H) and promote infiltration of CD8^+^ T cells with the enhanced expression of effector molecules (Figures 7I, 7J and S14D).

Next, we assessed the anti-metastatic effect of the combined therapeutic regimen by examining the spontaneous metastatic burden. We found that the combined regimen resulted in fewer lung metastases of 4T1 tumors (Figures 7K and 7L). Collectively, these data indicate that inhibiting Gal-1 could potentially counteract the immune-depressing environment, leading to more effective treatment when used alongside anti-PD-1 therapies.

## Discussion

**Figure 8:**
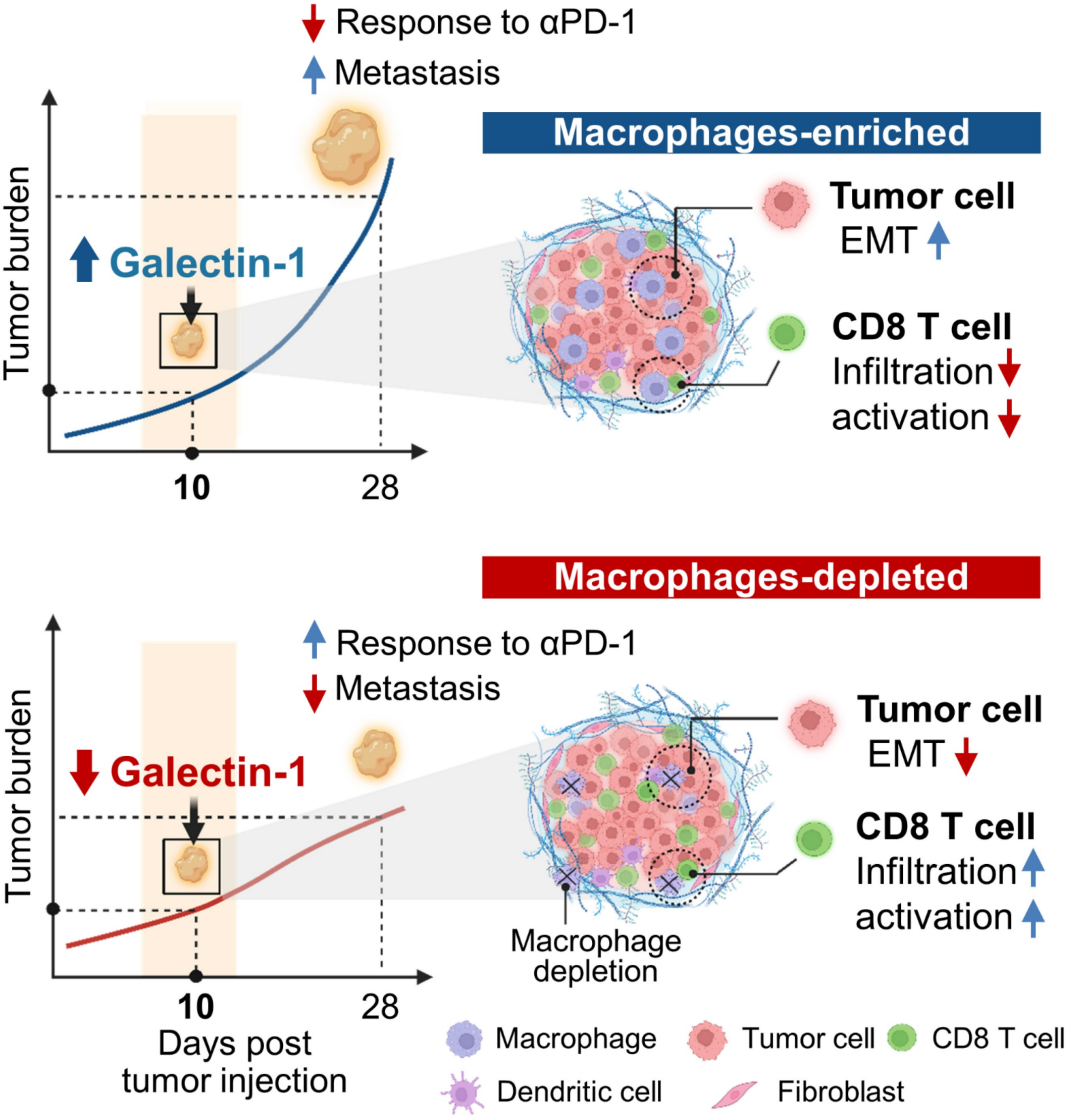
Schematic model for macrophage-mediated early tumor microenvironments.

In this study, we delineated the single cell-level molecular changes that occur in cancer cells and immune cells as a consequence of TAM depletion within early breast cancer TMEs. Although previous studies have reported TAM-mediated formation of conducive TMEs 21, 22, the associated key mediators, particularly in the early stages of tumor progression, have not yet been elucidated. Using single cell RNA-seq, we confirmed that TAM depletion induces the reorganization of tumor immune microenvironments and causes molecular changes in cancer cells, and identified Gal-1 as an immune modulatory target that can overcome TAM-mediated immunosuppressive TMEs (Figure 8).

Upon TAM depletion, the subtype proportion of cancer cells with high EMT signatures was reduced and activation of the mesenchymal program was attenuated. Our data suggested that the transition of cancer cells to mesenchymal-like states was promoted by TAMs during very early tumor stages, which is consistent with a recent study reporting that macrophages downregulate E-cadherin junctions in HER2^+^ early cancer cells 59. Notably, we found that *Igfbp5* had a significantly lower expression in cancer cells treated with the vehicle control compared to clodronate-treated cells and observed that TAM depletion markedly increased the level of *Igfbp5*. *Igfbp5* has been shown to be a tumor suppressor that inhibits migration, invasion, and EMT of cancer cells 60, 61. Overexpression of *Igfbp5* inhibited tumor growth and pulmonary metastases in different models including osteosarcoma 62 and melanoma 63. This suggests that *Igfbp5* may play a role in the observed decrease in the mesenchymal traits of cancer cells upon TAM depletion; however, further research is required to elucidate the mechanisms involved in the direct regulation of *Igfbp5* expression in cancer cells by TAMs.

We also observed several TAM-dependent changes in the immune cell population. Firstly, among the T cell populations, specific T cell populations that exhibited high *Satb1* and *CD8* expression were markedly affected. In the clodronate-treated tumor, the CD8_s1 cluster was significantly increased, while the CD8_s2 cluster was significantly decreased (Figure 3A). *Satb1* is closely related to immune tolerance and plays a key role in inhibiting PD-1 expression in T cells 64, 65. Therefore, the molecular changes in *Satb*^+^ T cells after TAM depletion may affect T cell-mediated antitumor immunity. These findings not only support previous studies showing that TAMs are important regulators of T cells during the formation of immunosuppressive TMEs, but also provide additional insights into the TAM-induced cellular and molecular alterations responsible for tumor immune escape during the early stages before exponential tumor growth 21.

To identify a novel candidate that is likely to play a fundamental role in driving TAM-mediated immunosuppressive TMEs, we compared the differentially expressed transcripts in T cells (as determined by scRNA-seq), of which Gal-1 was the most significantly downregulated in TAM-depleted tumors. The downregulation of Gal-1 expression was similarly demonstrated in B cells and myeloid cells upon TAM depletion. Gal-1 is produced by various cell types within the TME, including cancer cells, fibroblasts, endothelial cells, and immune cells. Both tumor- and stromal-derived Gal-1 contribute to tumor progression and metastasis by promoting cancer cell invasion 58, 66, 67, fibroblast activation 68, 69, and angiogenesis 70. Notably, Gal-1 has been recognized as a tumor-associated protein that contributes to the immune privilege of some tumors by impairing T cell effector function 57, 71. Mechanistically, Gal-1 modulates the survival and cytokine production of effector T cells 72, 73 and facilitates the expansion and immunosuppressive activity of Tregs 74, 75. Recently, the role of Gal-1 in the immunoregulatory functions of TAMs has also been described. For example, Gal-1 induces immunosuppressive M2 phenotype in TAMs with high expression of IDO and PDL1 76, 77 and TAMs-derived Gal-1 impedes CD8^+^ T cell recruitment to tumor 78. This is particularly important in a clinical setting because accumulating evidence has revealed that an abundance of TAMs with pro-tumorigenic functions promotes resistance to ICIs 16, 79, 80. TAM-derived chemokines implicated in T cell recruitment, such as *Cxcl9* and *Cxcl10*, were downregulated in ICI non-responsive tumors 19, 81, 82. Moreover, it has been found that TAMs induce a CD8^+^ T cell-excluded TME via direct and long-lasting interactions with CD8^+^ T cells 17 and the formation of fibrotic stroma 18, which inhibits T cell entry, resulting in poor clinical responses to ICIs. Thus, TAMs have attracted attention as a potent cellular target for improving therapeutic outcome to ICIs, and there are ongoing clinical trials utilizing TAM-targeting agents in combination with conventional ICIs to augment anti-cancer responses 21.

Our findings provide evidence that a Gal-1 blockade using OTX008 treatment successfully promoted antitumor CD8^+^ T cell responses, thereby sensitizing otherwise refractory breast cancers to anti-PD-1 treatment and attenuating tumor growth and lung metastases. These results are in accordance with previous data demonstrating the therapeutic potential of Gal-1 antibodies in combination with ICIs in head and neck cancer 83, further supporting the notion that Gal-1 inhibition is a promising strategy to potentiate the efficacy of ICIs in patients with various cancer types.

However, a limitation of our study it that although transcriptional changes following clodronate treatment revealed Gal-1 as a core gene in TAMs-dependent TME, we could not clearly demonstrate a direct relationship between TAMs and Gal-1 expression in T cells. The precise mechanisms by which TAMs regulate Gal-1 expression and subsequently induce immunosuppression remain to be determined. Additionally, we have mainly focused on the impact of Gal-1 on T cells to establish immunosuppressive TME. Future studies should explore functions of Gal-1 other than immunosuppression to fully assess its potential as a therapeutic target to revert TAMs-mediated niche, resulting in reduced tumor burden and increased response to conventional therapies. It would be also interesting to explore the contribution of different TAM subsets to tumor progression. More specifically, albeit clodronate used in this study depletes global types of TAMs, further comprehensive analysis for the landscape programmed by a specific type such as residential or recruiting TAMs could provide a basis for the development of macrophage-targeted therapeutic strategies.

In summary, this study elucidates TAM-dependent cellular and molecular crosstalk to induce a tumorigenic TME and increase the metastatic potential of breast cancers. We also confirmed Gal-1 to be an actionable molecule that can be inhibited to enhance intratumoral CD8^+^ T cell infiltration and reduce metastasis. Specific targeting of Gal-1 may reverse TAM-associated protumor niches without the adverse side effects caused by broad targeting of TAMs. This may provide a new avenue for the development of therapeutic strategies to overcome immunosuppression, with profound implications for immunotherapeutic approaches.

## Supplementary Material

Supplementary figures and table 2.

Supplementary table 1.

## Abbreviations

TAM: tumor-associated macrophage
TME: tumor microenvironment
EMT: epithelial-to-mesenchymal transition
Gal-1: galectin-1
MDSC: myeloid-derived suppressor cell
Tregs: regulatory T cell
ICI: immune checkpoint inhibitor
scRNA-seq: single-cell RNA sequencing
BMDM: bone marrow-derived macrophage
UMAP: uniform manifold approximation and projection
DEG: differentially expressed gene
TES: tumor explant supernatant
TUNEL: terminal deoxynucleotidyl transferase dUTP nick end labeling

## References

[1] Vitale I, Shema E, Loi S, Galluzzi L (2021). Intratumoral heterogeneity in cancer progression and response to immunotherapy. Nat Med.

[2] Patel AP, Tirosh I, Trombetta JJ, Shalek AK, Gillespie SM, Wakimoto H (2014). Single-cell RNA-seq highlights intratumoral heterogeneity in primary glioblastoma. Science.

[3] Vitale I, Sistigu A, Manic G, Rudqvist N-P, Trajanoski Z, Galluzzi L (2019). Mutational and antigenic landscape in tumor progression and cancer immunotherapy. Trends Cell Biol.

[4] Dagogo-Jack I, Shaw AT (2018). Tumour heterogeneity and resistance to cancer therapies. Nat Rev Clin Oncol.

[5] Junttila MR, De Sauvage FJ (2013). Influence of tumour micro-environment heterogeneity on therapeutic response. Nature.

[6] Han B, Meng X, Wu P, Li Z, Li S, Zhang Y (2020). ATRX/EZH2 complex epigenetically regulates FADD/PARP1 axis, contributing to TMZ resistance in glioma. Theranostics.

[7] Qian B-Z, Li J, Zhang H, Kitamura T, Zhang J, Campion LR (2011). CCL2 recruits inflammatory monocytes to facilitate breast-tumour metastasis. Nature.

[8] Kim H, Chung H, Kim J, Choi DH, Shin Y, Kang YG (2019). Macrophages-triggered sequential remodeling of endothelium-interstitial matrix to form pre-metastatic niche in microfluidic tumor microenvironment. Adv Sci.

[9] Harney AS, Arwert EN, Entenberg D, Wang Y, Guo P, Qian B-Z (2015). Real-time imaging reveals local, transient vascular permeability, and tumor cell intravasation stimulated by TIE2hi macrophage-derived VEGFA. Cancer Discov.

[10] De Palma M, Venneri MA, Galli R, Sergi LS, Politi LS, Sampaolesi M (2005). Tie2 identifies a hematopoietic lineage of proangiogenic monocytes required for tumor vessel formation and a mesenchymal population of pericyte progenitors. Cancer Cell.

[11] Pagano E, Elias JE, Schneditz G, Saveljeva S, Holland LM, Borrelli F (2022). Activation of the GPR35 pathway drives angiogenesis in the tumour microenvironment. Gut.

[12] Qian B-Z, Pollard JW (2010). Macrophage diversity enhances tumor progression and metastasis. Cell.

[13] DeNardo DG, Brennan DJ, Rexhepaj E, Ruffell B, Shiao SL, Madden SF (2011). Leukocyte complexity predicts breast cancer survival and functionally regulates response to chemotherapy. Cancer Discov.

[14] Na YR, Kwon JW, Chung H, Song J, Jung D, Quan H (2020). Protein kinase A catalytic subunit is a molecular switch that promotes the pro-tumoral function of macrophages. Cell Rep.

[15] Kaneda MM, Messer KS, Ralainirina N, Li H, Leem CJ, Gorjestani S (2016). PI3Kγ is a molecular switch that controls immune suppression. Nature.

[16] Arlauckas SP, Garris CS, Kohler RH, Kitaoka M, Cuccarese MF, Yang KS (2017). *In vivo* imaging reveals a tumor-associated macrophage-mediated resistance pathway in anti-PD-1 therapy. Sci Transl Med.

[17] Peranzoni E, Lemoine J, Vimeux L, Feuillet V, Barrin S, Kantari-Mimoun C (2018). Macrophages impede CD8 T cells from reaching tumor cells and limit the efficacy of anti-PD-1 treatment. PNAS.

[18] Quaranta V, Rainer C, Nielsen SR, Raymant ML, Ahmed MS, Engle DD (2018). Macrophage-derived granulin drives resistance to immune checkpoint inhibition in metastatic pancreatic cancer. Cancer Res.

[19] Pascual-García M, Bonfill-Teixidor E, Planas-Rigol E, Rubio-Perez C, Iurlaro R, Arias A (2019). LIF regulates CXCL9 in tumor-associated macrophages and prevents CD8+ T cell tumor-infiltration impairing anti-PD1 therapy. Nat Commun.

[20] Cassetta L, Pollard JW (2018). Targeting macrophages: therapeutic approaches in cancer. Nat Rev Drug Discov.

[21] DeNardo DG, Ruffell B (2019). Macrophages as regulators of tumour immunity and immunotherapy. Nat Rev Immunol.

[22] Bejarano L, Jordāo MJ, Joyce JA (2021). Therapeutic targeting of the tumor microenvironment. Cancer Discov.

[23] Zeisberger S, Odermatt B, Marty C, Zehnder-Fjällman A, Ballmer-Hofer K, Schwendener R (2006). Clodronate-liposome-mediated depletion of tumour-associated macrophages: a new and highly effective antiangiogenic therapy approach. Br J Cancer.

[24] Qian B, Deng Y, Im JH, Muschel RJ, Zou Y, Li J (2009). A distinct macrophage population mediates metastatic breast cancer cell extravasation, establishment and growth. PloS One.

[25] Pyonteck SM, Akkari L, Schuhmacher AJ, Bowman RL, Sevenich L, Quail DF (2013). CSF-1R inhibition alters macrophage polarization and blocks glioma progression. Nat Med.

[26] Xu J, Escamilla J, Mok S, David J, Priceman S, West B (2013). CSF1R signaling blockade stanches tumor-infiltrating myeloid cells and improves the efficacy of radiotherapy in prostate cancer. Cancer Res.

[27] Mitchem JB, Brennan DJ, Knolhoff BL, Belt BA, Zhu Y, Sanford DE (2013). Targeting tumor-infiltrating macrophages decreases tumor-initiating cells, relieves immunosuppression, and improves chemotherapeutic responses. Cancer Res.

[28] Sanford DE, Belt BA, Panni RZ, Mayer A, Deshpande AD, Carpenter D (2013). Inflammatory monocyte mobilization decreases patient survival in pancreatic cancer: a role for targeting the CCL2/CCR2 axis. Clin. Cancer Res.

[29] Kalbasi A, Komar C, Tooker GM, Liu M, Lee JW, Gladney WL (2017). Tumor-derived CCL2 mediates resistance to radiotherapy in pancreatic ductal adenocarcinoma. Clin Cancer Res.

[30] Nywening TM, Wang-Gillam A, Sanford DE, Belt BA, Panni RZ, Cusworth BM (2016). Targeting tumour-associated macrophages with CCR2 inhibition in combination with FOLFIRINOX in patients with borderline resectable and locally advanced pancreatic cancer: a single-centre, open-label, dose-finding, non-randomised, phase 1b trial. Lancet Oncol.

[31] Halama N, Zoernig I, Berthel A, Kahlert C, Klupp F, Suarez-Carmona M (2016). Tumoral immune cell exploitation in colorectal cancer metastases can be targeted effectively by anti-CCR5 therapy in cancer patients. Cancer Cell.

[32] Walens A, DiMarco AV, Lupo R, Kroger BR, Damrauer JS, Alvarez JV (2019). CCL5 promotes breast cancer recurrence through macrophage recruitment in residual tumors. Elife.

[33] Huang R, Wang S, Wang N, Zheng Y, Zhou J, Yang B (2020). CCL5 derived from tumor-associated macrophages promotes prostate cancer stem cells and metastasis via activating β-catenin/STAT3 signaling. Cell Death Dis.

[34] Kaneda MM, Cappello P, Nguyen AV, Ralainirina N, Hardamon CR, Foubert P (2016). Macrophage PI3Kγ drives pancreatic ductal adenocarcinoma progression. Cancer Discov.

[35] De Henau O, Rausch M, Winkler D, Campesato LF, Liu C, Cymerman DH (2016). Overcoming resistance to checkpoint blockade therapy by targeting PI3Kγ in myeloid cells. Nature.

[36] Sai J, Owens P, Novitskiy SV, Hawkins OE, Vilgelm AE, Yang J (2017). PI3K inhibition reduces mammary tumor growth and facilitates antitumor immunity and anti-PD1 responses. Clin Cancer Res.

[37] Cassetta L, Fragkogianni S, Sims AH, Swierczak A, Forrester LM, Zhang H (2019). Human tumor-associated macrophage and monocyte transcriptional landscapes reveal cancer-specific reprogramming, biomarkers, and therapeutic targets. Cancer Cell.

[38] Qian J, Olbrecht S, Boeckx B, Vos H, Laoui D, Etlioglu E (2020). A pan-cancer blueprint of the heterogeneous tumor microenvironment revealed by single-cell profiling. Cell Res.

[39] Loyher P-L, Hamon P, Laviron M, Meghraoui-Kheddar A, Goncalves E, Deng Z (2018). Macrophages of distinct origins contribute to tumor development in the lung. J Exp Med.

[40] Müller S, Kohanbash G, Liu SJ, Alvarado B, Carrera D, Bhaduri A (2017). Single-cell profiling of human gliomas reveals macrophage ontogeny as a basis for regional differences in macrophage activation in the tumor microenvironment. Genome Biol.

[41] Pienta KJ, Machiels J-P, Schrijvers D, Alekseev B, Shkolnik M, Crabb SJ (2013). Phase 2 study of carlumab (CNTO 888), a human monoclonal antibody against CC-chemokine ligand 2 (CCL2), in metastatic castration-resistant prostate cancer. Invest. New Drugs.

[42] Ries CH, Cannarile MA, Hoves S, Benz J, Wartha K, Runza V (2014). Targeting tumor-associated macrophages with anti-CSF-1R antibody reveals a strategy for cancer therapy. Cancer cell.

[43] Cannarile MA, Weisser M, Jacob W, Jegg A-M, Ries CH, Rüttinger D (2017). Colony-stimulating factor 1 receptor (CSF1R) inhibitors in cancer therapy. J Immunother Cancer.

[44] Kaneda MM, Cappello P, Nguyen AV, Ralainirina N, Hardamon CR, Foubert P (2016). Macrophage PI3Kγ Drives Pancreatic Ductal Adenocarcinoma ProgressionPI3Kγ Is a Novel Therapeutic Target in Pancreas Cancer. Cancer Discov.

[45] Ianevski A, Giri AK, Aittokallio T (2022). Fully-automated and ultra-fast cell-type identification using specific marker combinations from single-cell transcriptomic data. Nature Commun.

[46] Finak G, McDavid A, Yajima M, Deng J, Gersuk V, Shalek AK (2015). MAST: a flexible statistical framework for assessing transcriptional changes and characterizing heterogeneity in single-cell RNA sequencing data. Genome Biol.

[47] Cao J, Spielmann M, Qiu X, Huang X, Ibrahim DM, Hill AJ (2019). The single-cell transcriptional landscape of mammalian organogenesis. Nature.

[48] Browaeys R, Saelens W, Saeys Y (2020). NicheNet: modeling intercellular communication by linking ligands to target genes. Nat Methods.

[49] Bassez A, Vos H, Van Dyck L, Floris G, Arijs I, Desmedt C (2021). A single-cell map of intratumoral changes during anti-PD1 treatment of patients with breast cancer. Nat Med.

[50] Lou Y, Diao L, Cuentas ERP, Denning WL, Chen L, Fan YH (2016). Epithelial-mesenchymal transition is associated with a distinct tumor microenvironment including elevation of inflammatory signals and multiple immune checkpoints in lung adenocarcinoma. Clin Cancer Res.

[51] Li Z, Meng X, Wu P, Zha C, Han B, Li L (2021). Glioblastoma cell-derived lncRNA-containing exosomes induce microglia to produce complement C5, promoting chemotherapy resistance. Cancer Immunol Res.

[52] Muhammad N, Bhattacharya S, Steele R, Phillips N, Ray RB (2017). Involvement of c-Fos in the Promotion of Cancer Stem-like Cell Properties in Head and Neck Squamous Cell Carcinomac-Fos in the Enhancement of Cancer Stem-like Properties. Clin Cancer Res.

[53] Wu W-S, You R-I, Cheng C-C, Lee M-C, Lin T-Y, Hu C-T (2017). Snail collaborates with EGR-1 and SP-1 to directly activate transcription of MMP 9 and ZEB1. Sci Rep.

[54] Shao X, Liao J, Li C, Lu X, Cheng J, Fan X (2021). CellTalkDB: a manually curated database of ligand-receptor interactions in humans and mice. Brief Bioinformatics.

[55] Nielsen MI, Stegmayr J, Grant OC, Yang Z, Nilsson UJ, Boos I (2018). Galectin binding to cells and glycoproteins with genetically modified glycosylation reveals galectin-glycan specificities in a natural context. J Biol Chem.

[56] Delord J-P, Awada A, Raymond E, Lokiec F, Herait P, Rezai K (2013). Abstract A72: A first-in-man Phase I study of the galectin-1 (gal-1) inhibitor OTX008 given subcutaneously as a single agent to patients with advanced solid tumors. AACR.

[57] Rubinstein N, Alvarez M, Zwirner NW, Toscano MA, Ilarregui JM, Bravo A (2004). Targeted inhibition of galectin-1 gene expression in tumor cells results in heightened T cell-mediated rejection: a potential mechanism of tumor-immune privilege. Cancer Cell.

[58] Banh A, Zhang J, Cao H, Bouley DM, Kwok S, Kong C (2011). Tumor galectin-1 mediates tumor growth and metastasis through regulation of T-cell apoptosis. Cancer Res.

[59] Linde N, Casanova-Acebes M, Sosa MS, Mortha A, Rahman A, Farias E (2018). Macrophages orchestrate breast cancer early dissemination and metastasis. Nat Commun.

[60] Sureshbabu A, Okajima H, Yamanaka D, Tonner E, Shastri S, Maycock J (2012). IGFBP5 induces cell adhesion, increases cell survival and inhibits cell migration in MCF-7 human breast cancer cells. J Cell Sci.

[61] Zhang L, Li W, Cao L, Xu J, Qian Y, Chen H (2019). PKNOX2 suppresses gastric cancer through the transcriptional activation of IGFBP5 and p53. Oncogene.

[62] Su Y, Wagner E, Luo Q, Huang J, Chen L, He B (2011). Insulin-like growth factor binding protein 5 suppresses tumor growth and metastasis of human osteosarcoma. Oncogene.

[63] Wang J, Ding N, Li Y, Cheng H, Wang D, Yang Q (2015). Insulin-like growth factor binding protein 5 (IGFBP5) functions as a tumor suppressor in human melanoma cells. Oncotarget.

[64] Kondo M, Tanaka Y, Kuwabara T, Naito T, Kohwi-Shigematsu T, Watanabe A (2016). SATB1 plays a critical role in establishment of immune tolerance. J Immunol.

[65] Stephen TL, Payne KK, Chaurio RA, Allegrezza MJ, Zhu H, Perez-Sanz J (2017). SATB1 expression governs epigenetic repression of PD-1 in tumor-reactive T cells. Immunity.

[66] Martínez-Bosch N, Fernández-Barrena MG, Moreno M, Ortiz-Zapater E, Munné-Collado J, Iglesias M (2014). Galectin-1 drives pancreatic carcinogenesis through stroma remodeling and Hedgehog signaling activation. Cancer Res.

[67] Chung L-Y, Tang S-J, Sun G-H, Chou T-Y, Yeh T-S, Yu S-L (2012). Galectin-1 promotes lung cancer progression and chemoresistance by upregulating p38 MAPK, ERK, and cyclooxygenase-2. Clin Cancer Res.

[68] Orozco CA, Martinez-Bosch N, Guerrero PE, Vinaixa J, Dalotto-Moreno T, Iglesias M (2018). Targeting galectin-1 inhibits pancreatic cancer progression by modulating tumor-stroma crosstalk. PNAS.

[69] Wu M-H, Hong H-C, Hong T-M, Chiang W-F, Jin Y-T, Chen Y-L (2011). Targeting galectin-1 in carcinoma-associated fibroblasts inhibits oral squamous cell carcinoma metastasis by downregulating MCP-1/CCL2 expression. Clin Cancer Res.

[70] Thijssen VL, Postel R, Brandwijk RJ, Dings RP, Nesmelova I, Satijn S (2006). Galectin-1 is essential in tumor angiogenesis and is a target for antiangiogenesis therapy. PNAS.

[71] Dalotto-Moreno T, Croci DO, Cerliani JP, Martinez-Allo VC, Dergan-Dylon S, Méndez-Huergo SP (2013). Targeting galectin-1 overcomes breast cancer-associated immunosuppression and prevents metastatic disease. Cancer Res.

[72] Toscano MA, Bianco GA, Ilarregui JM, Croci DO, Correale J, Hernandez JD (2007). Differential glycosylation of TH1, TH2 and TH-17 effector cells selectively regulates susceptibility to cell death. Nat Immunol.

[73] Ilarregui JM, Croci DO, Bianco GA, Toscano MA, Salatino M, Vermeulen ME (2009). Tolerogenic signals delivered by dendritic cells to T cells through a galectin-1-driven immunoregulatory circuit involving interleukin 27 and interleukin 10. Nat Immunol.

[74] Cagnoni AJ, Giribaldi ML, Blidner AG, Cutine AM, Gatto SG, Morales RM (2021). Galectin-1 fosters an immunosuppressive microenvironment in colorectal cancer by reprogramming CD8+ regulatory T cells. PNAS.

[75] Garín MI, Chu C-C, Golshayan D, Cernuda-Morollón E, Wait R, Lechler RI (2007). Galectin-1: a key effector of regulation mediated by CD4+ CD25+ T cells. Blood.

[76] Rudjord-Levann AM, Ye Z, Hafkenscheid L, Horn S, Wiegertjes R, Nielsen MA (2023). Galectin-1 induces a tumor-associated macrophage phenotype and upregulates indoleamine 2, 3-dioxygenase-1. Iscience.

[77] Chen Q, Han B, Meng X, Duan C, Yang C, Wu Z (2019). Immunogenomic analysis reveals LGALS1 contributes to the immune heterogeneity and immunosuppression in glioma. Int J Cancer.

[78] Tan J, Fan W, Liu T, Zhu B, Liu Y, Wang S (2023). TREM2+ macrophages suppress CD8+ T-cell infiltration after transarterial chemoembolisation in hepatocellular carcinoma. J Hepatol.

[79] Muraoka D, Seo N, Hayashi T, Tahara Y, Fujii K, Tawara I (2019). Antigen delivery targeted to tumor-associated macrophages overcomes tumor immune resistance. J Clin Investig.

[80] Wei Z, Zhang X, Yong T, Bie N, Zhan G, Li X (2021). Boosting anti-PD-1 therapy with metformin-loaded macrophage-derived microparticles. Nat Commun.

[81] Qu Y, Wen J, Thomas G, Yang W, Prior W, He W (2020). Baseline frequency of inflammatory Cxcl9-Expressing tumor-associated macrophages predicts response to Avelumab treatment. Cell Rep.

[82] House IG, Savas P, Lai J, Chen AX, Oliver AJ, Teo ZL (2020). Macrophage-derived CXCL9 and CXCL10 are required for antitumor immune responses following immune checkpoint blockade. Clin Cancer Res.

[83] Nambiar DK, Aguilera T, Cao H, Kwok S, Kong C, Bloomstein J (2019). Galectin-1-driven T cell exclusion in the tumor endothelium promotes immunotherapy resistance. J Clin Investig.

