# Supplementary material for: Comprehensive characterization of early-programmed tumor microenvironment by tumor-associated macrophages reveals galectin-1 as an immune modulatory target in breast cancer
Source: Theranostics. 2024 Jan 1;14(2):843–60. doi: 10.7150/thno.88917 (PMC10758049; doi:10.7150/thno.88917)
Supplement: Supplementary file 1 — Supplementary figures and table 2. [file thnov14p0843s1.pdf]

Supplementary Material

Comprehensive characterization of early-programmed tumor microenvironment by tumor-associated macrophages reveals galectin-1 as an immune modulatory target in breast cancer

Hyewon Chung[1,2], Park Gyu-mi[1,3], Yi Rang Na[4], Yun-Sang Lee[5,6], Hongyoon Choi[5,6]*, Seung Hyeok Seok[1,3,7]*

[1]Macrophage Lab, Department of Microbiology and Immunology, and Institute of Endemic Disease, Seoul National University College of Medicine, Seoul 110-799, South Korea

[2]Institute of Endemic Diseases, Seoul National University Medical Research Center (SNUMRC), Seoul, Republic of Korea

[3]Department of Biomedical Sciences and Seoul National University College of Medicine, Seoul, Republic of Korea

[4]Transdisciplinary Department of Medicine and Advanced Technology, Seoul National University Hospital, Seoul, South Korea

[5]Department of Nuclear Medicine, Seoul National University Hospital, Seoul, Republic of Korea

[6]Department of Nuclear Medicine, Seoul National University College of Medicine, Seoul, Republic of Korea

[7]Cancer Research Institute, Seoul National University, Seoul, South Korea

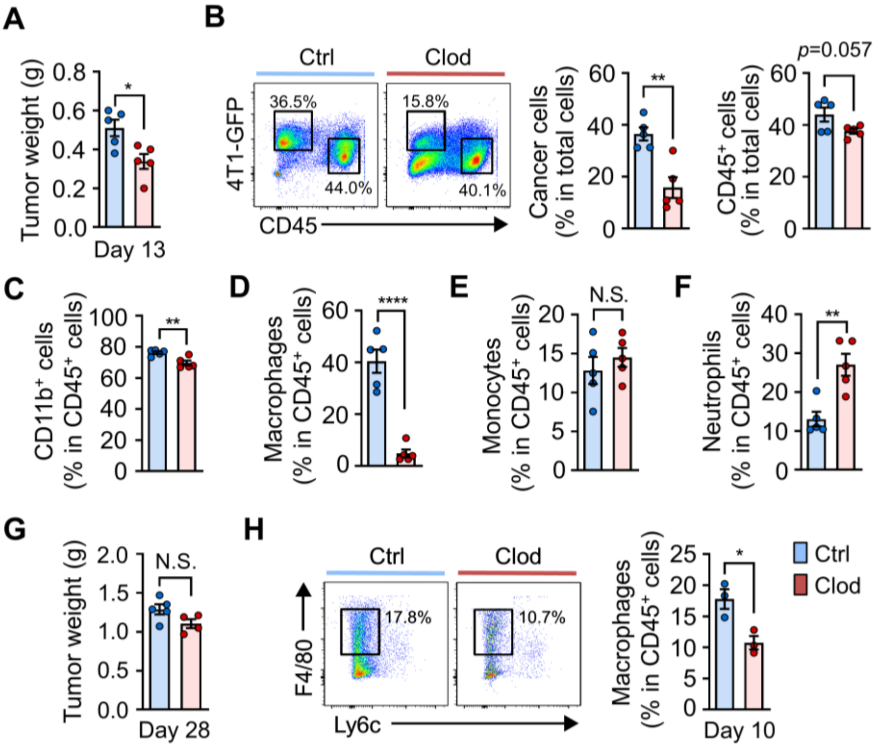

Figure S1. Characterization of immune populations after clodronate treatment. (A) Tumor weight in 4T1 tumor-bearing mice after vehicle (control, n = 5 mice) or clodronate (clod, n = 5 mice) treatment every 2–3 days for 10 days. (B) Representative flow plots (left) and percentages of cancer cells (4T1-GFP cells) and CD45$^+$ cells (right) in 4T1-bearing mice treated with clod or vehicle (n = 5 mice per group). (C-F) Flow cytometric quantification of CD11b$^+$ myeloid cells (C), macrophages (D), monocytes (E), and neutrophils (F) between the two experimental groups. Data are expressed as the percentage of total CD45$^+$ cells. (G) Tumor weights in 4T1 tumor-bearing mice transiently treated with clod (n = 4 mice) or vehicle (n = 5 mice) at day 28. (H) Representative flow plots (left) and flow cytometric quantification of macrophages (CD45$^+$CD11b$^+$Gr1$^-$F4/80$^+$Ly6c$^-$) in tumors treated with clod (n = 3 mice) or vehicle (n = 3 mice) at day 10. All data represented as mean ± S.E.M. Statistical significance was determined by two-tailed *t*-tests. **P* < 0.05, ***P* < 0.01 and *****P* < 0.0001. N.S., nonsignificant.

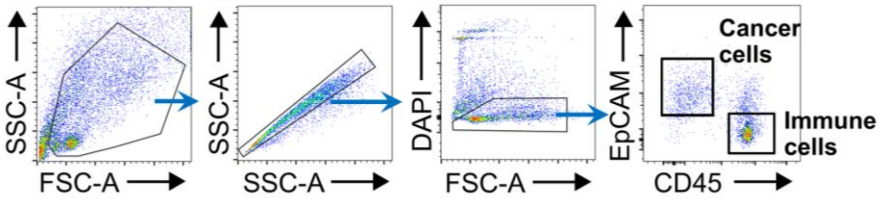

Figure S2. Gating strategy for sorting of cancer cells and immune cells from tumor masses after treatment with clodronate (Clod) or vehicle (Ctrl) for scRNA-seq analysis. Gating strategy used to sort cancer cells ($CD45^-EpCAM^+$) and immune cells ($CD45^+EpCAM^-$) from 4T1 tumor samples for scRNA-seq analysis.

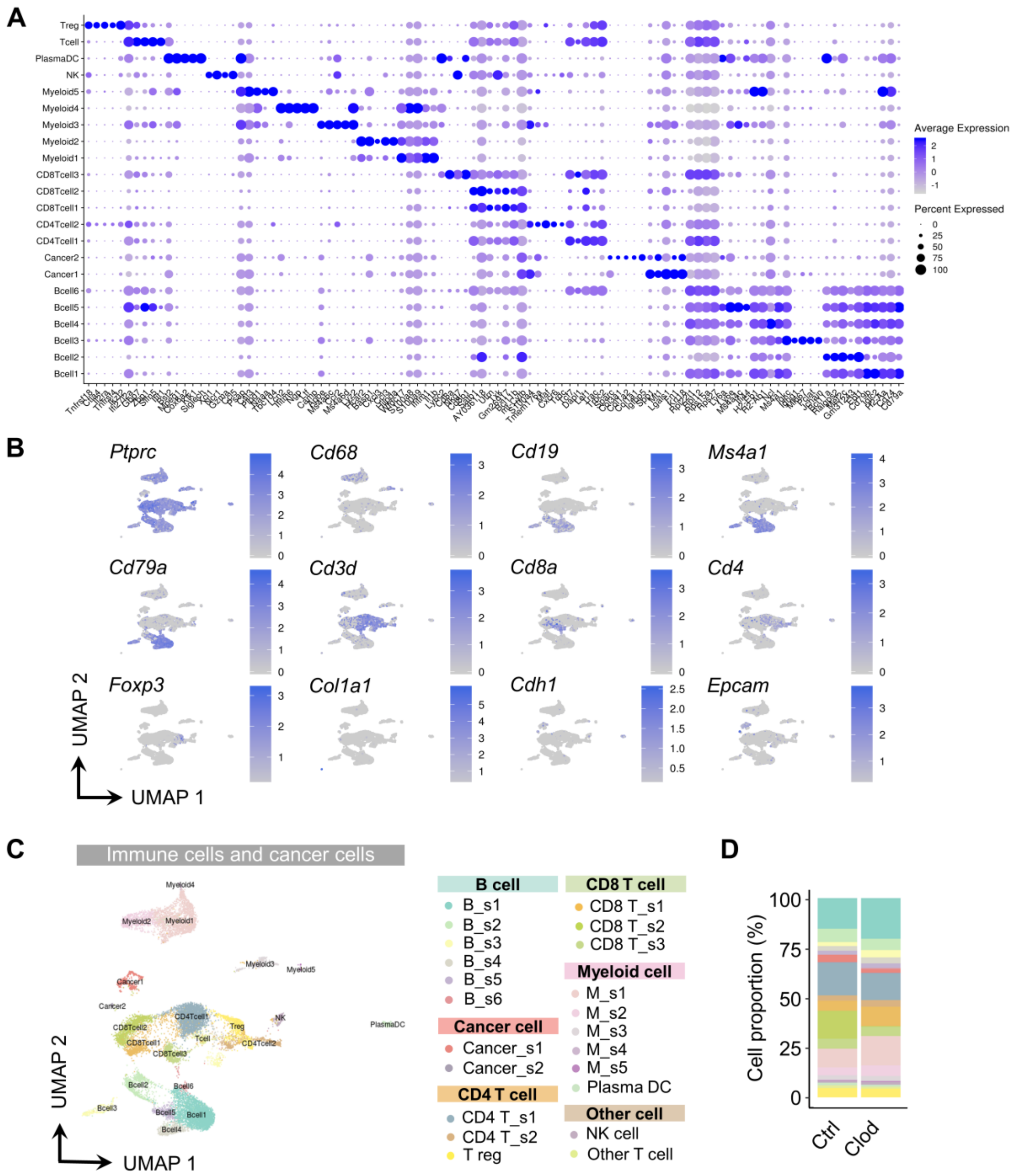

Figure S3. Cell subtypes and markers. (A) scRNA-seq data were clustered and markers were identified. The gene expression of markers is represented by a dotplot. (B) Gene expression of key markers of immune cells and cancer cells is represented. These known markers were used to define the cell types of clusters. (C) UMAP of total cells, including immune cells and cancer cells, colored to represent the 22 annotated cell type clusters. (D) Proportions of each subcluster in the two experimental groups.

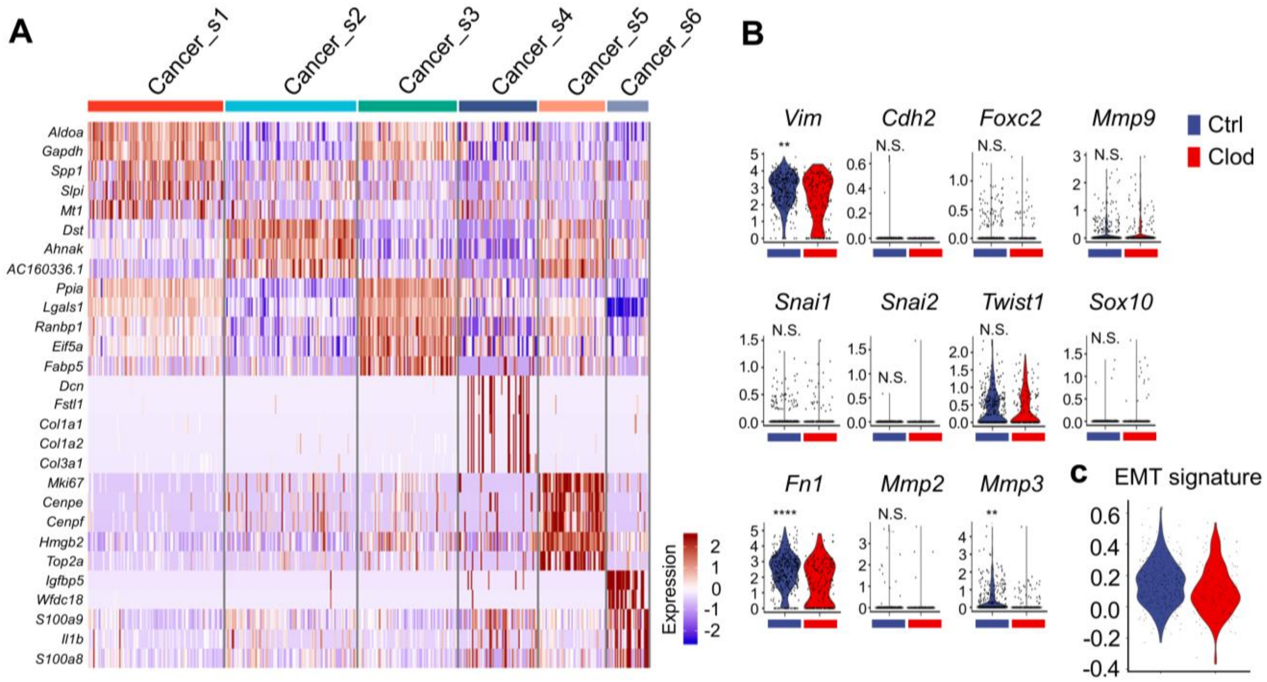

Figure S4. Subcluster of cancer cells and EMT signature. (A) Heatmap displaying the top marker genes for each cancer cell subcluster. (B) Violin plots showing the expression of selected mesenchymal feature-related genes in cancer cells following treatment with clodronate (clod) or vehicle (ctrl). (C) Violin plots showing EMT signature scores in cancer cells from ctrl- and clod-treated tumors. Significance of differential expression was determined by Mann Whitney tests. $^{**}P < 0.01$ and $^{****}P < 0.0001$. N.S., nonsignificant.

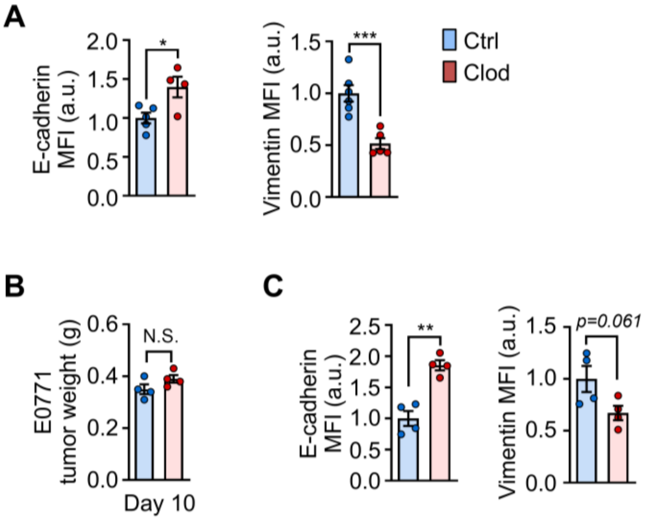

Figure S5. EMT phenotypes altered by clodronate treatment. (A) Flow cytometric analysis of the expression of E-cadherin (left) and vimentin (right) in cancer cells ($EpCAM^{+}CD45^{-}$), expressed as the mean fluorescence intensity (MFI) in 4T1 tumor (n = 4~5 mice per group). (B) Tumor weight in E0771-tumor bearing mice after vehicle (control, n = 4 mice) or clodronate (clod, n = 4 mice) treatment every 2–3 days for 7 days. (C) Flow cytometric analysis of the expression of E-cadherin (left) and vimentin (right) in cancer cells, expressed as MFI in E0771 tumor. *P < 0.05, **P < 0.01 and ***P < 0.001. N.S., nonsignificant.

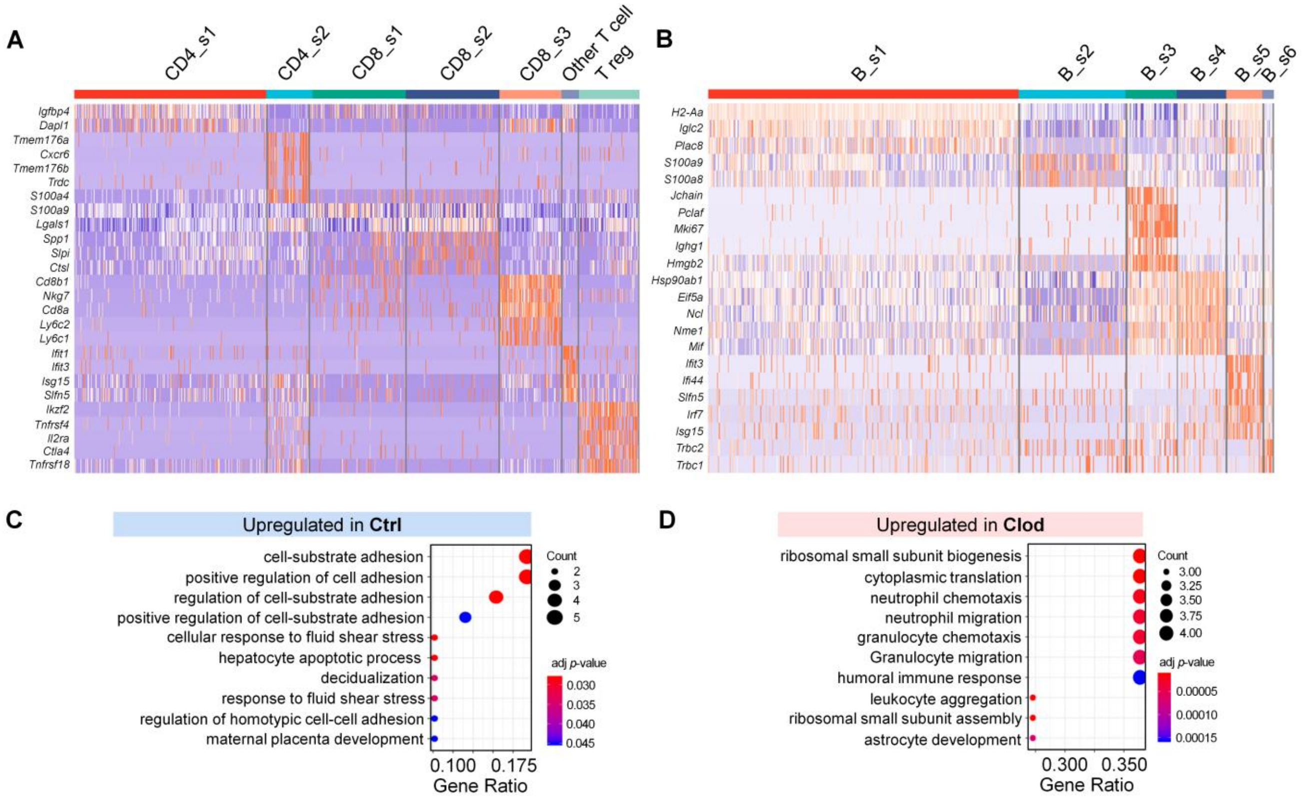

Figure S6. Markers of lymphocytes subclusters and GO analysis related to TAM depletion. (A and B) The marker genes of T cell subclusters (A) and B cell subclusters (B) were represented by heatmaps. (C and D) Enriched GO functions of upregulated genes in B cells from control (ctrl) (C) and clodronate (clod) groups (D).

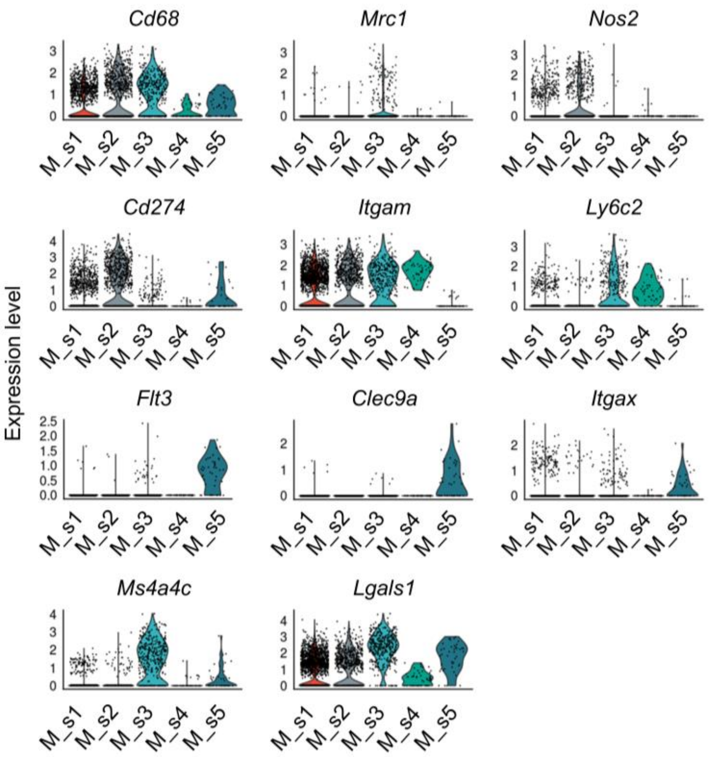

Figure S7. Markers of myeloid cells subclusters. Violin plots displaying the normalized expression levels of key marker genes across the five myeloid cell subclusters.

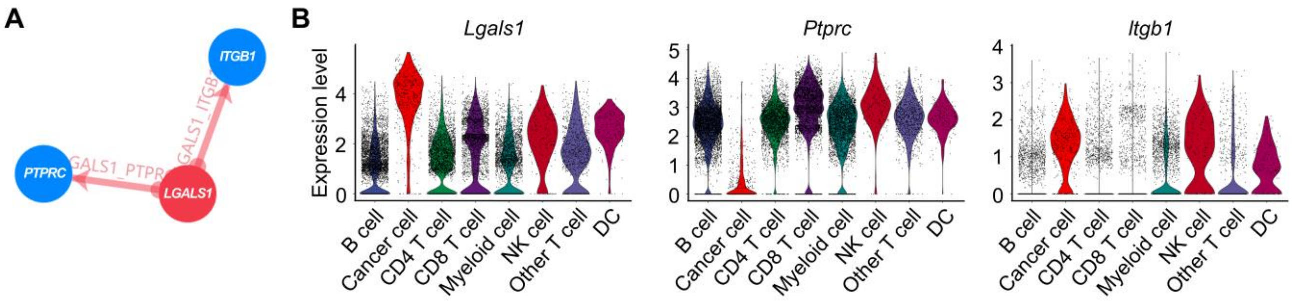

Figure S8. Receptor-ligand interaction of *LGALS1*. (A) The human receptor-ligand database indicated that *LGALS1* predominantly interacts with *PTPRC* and *ITGB1*. (B) Violin plots displaying the normalized expression levels of *Lgals1*, *Ptprc,* and *Itgb1* across the cell type clusters.

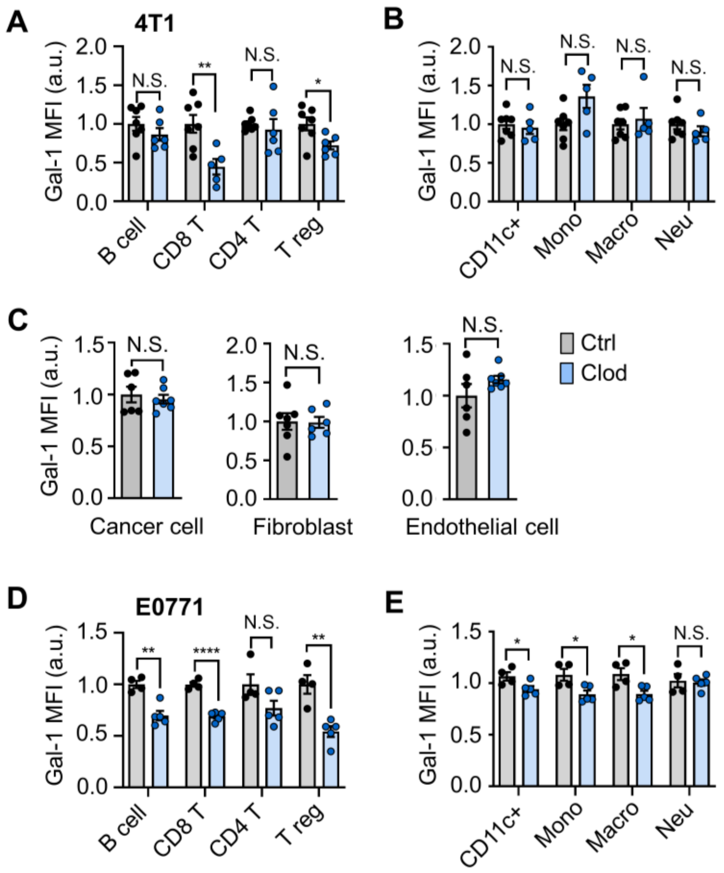

Figure S9. Comparison of Ga1-1 expression following clodronate treatment. (A and B) Flow cytometric analysis of Gal-1 staining in the indicated lymphoid cells (A) and myeloid cells (B) from 4T1 tumors treated with either clodronate (clod, n = 5~7) or vehicle (ctrl, n = 6~7). (C) Flow cytometric analysis of Gal-1 staining in the cancer cells, fibroblasts and endothelial cells. (D and E) Flow cytometric analysis of Gal-1 staining in the indicated lymphoid cells (D) and myeloid cells (E) from E0771 tumors treated with either clod (n = 5) or vehicle (n = 4). All data represented as mean ± S.E.M. Statistical significance was determined by two-tailed *t*-tests. *$P < 0.05$, **$P < 0.01$ and ****$P < 0.0001$. N.S., nonsignificant.

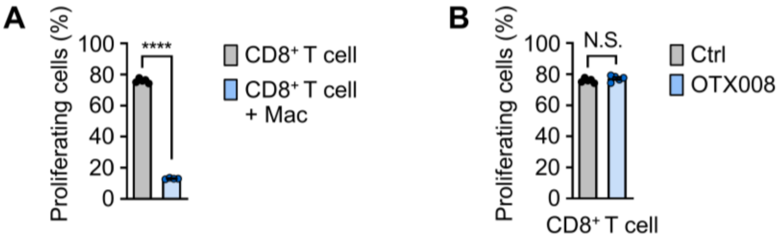

Figure S10. Effects of co-culture with macrophages or OTX008 treatment in CD8$^+$ T cell proliferation. (A) Proliferation of CD8$^+$ T cells alone or in co-culture with macrophages (Mac) (n = 5 per group). (B) Proliferation of CD8$^+$ T cells in the presence of vehicle or OTX008 (10 μM) (n = 5 per group). All data represented as mean ± S.E.M. Statistical significance was determined by two-tailed *t*-test. ****P < 0.0001. N.S., nonsignificant.

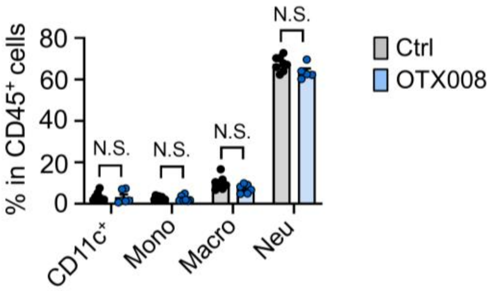

Figure S11. OTX008 treatment does not affect the proportion of myeloid cells. Flow cytometric quantification of $CD11b^+$ myeloid cells in tumors treated with OTX008 (n = 5–6 mice) or vehicle (n = 7–8 mice). Data are expressed as the percentage of $CD45^+$ cells. All data represented as mean ± S.E.M. Statistical significance was determined by two-tailed *t*-tests. N.S., nonsignificant.

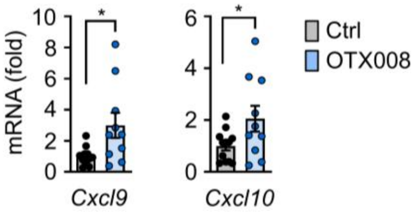

Figure S12. Pharmacological inhibition of Gal-1 using OTX008 increases the expression of *Cxcl9* and *Cxcl10*. mRNA expression of *Cxcl9* and *Cxcl10* in tumor tissues from 4T1 tumor-bearing mice treated with OTX008 (n = 10 mice) or vehicle (n = 11 mice). All data represented as mean ± S.E.M. Statistical significance was determined by two-tailed *t*-tests. $*P < 0.05$

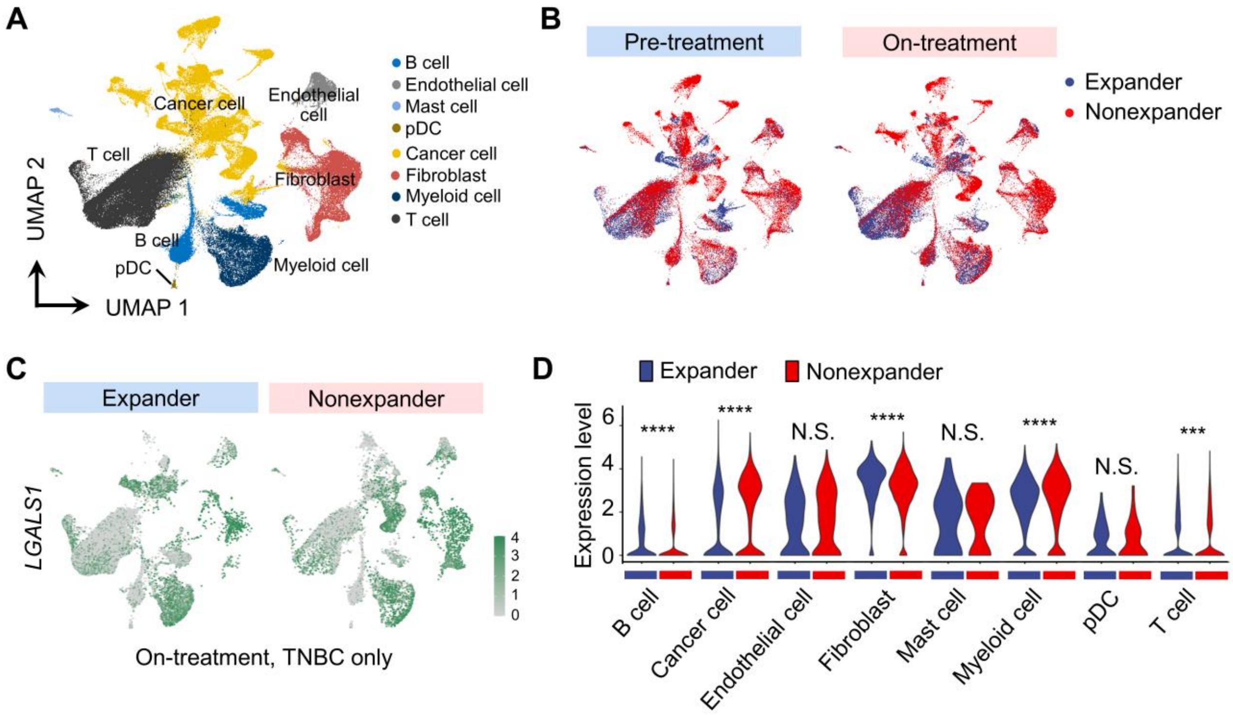

Figure S13. Gal-1 expression in scRNA-seq data of human breast cancer treated with anti-PD-1. scRNA-seq data of human breast cancer tissues during anti-PD-1 treatment were used to analyze Gal-1 expression. (A) UMAP plot of malignant and non-malignant cells, colored based on broad cell type. (B) UMAP plots of malignant and non-malignant cells for paired samples (pre- versus on-treatment), colored based on anti-PD-1 response (T cell-expander versus nonexpander). (C) UMAP plots of *LGALS1* expression in T cell-expanding versus -nonexpanding on-treatment triple-negative breast cancer (TNBC) samples. (D) Violin plots comparing expression distributions of *LGALS1* across the cell types from T cell-expanders versus nonexpanders. T cell-nonexpanders exhibited relatively higher levels of *LGALS1* expression in cancer cells, myeloid cells and T cells compared with T cell-expanders. However, fibroblasts exhibited relatively lower *LGALS1* expression in T cell-nonexpanders. Significance of differential expression was determined by Mann Whitney tests. ***$P < 0.001$ and ****$P < 0.0001$. N.S., nonsignificant.

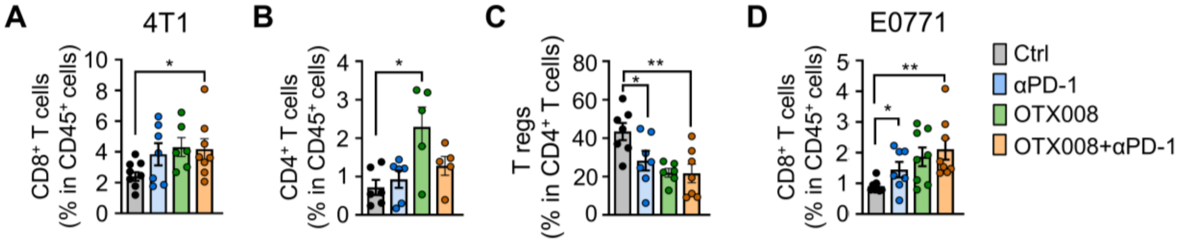

Figure S14. Combined Gal-1 and PD-1 blockade induces increased infiltration of T cells and reduces the proportion of Tregs. (A-C) Flow cytometric quantification of tumor-infiltrating $CD8^+$ T cells (A), $CD4^+$ T cells (B), and Tregs (C) in 4T1 tumor-bearing mice as described in Figure 8. (D) Flow cytometric quantification of tumor-infiltrating $CD8^+$ T cells in E0771 tumor-bearing mice. Data are expressed as the percentage of $CD45^+$ cells, except for Tregs, which are expressed as the percentage of total $CD4^+$ T cells. All data represented as mean ± S.E.M. Statistical significance was determined by two-tailed *t*-tests. $*P < 0.05$ and $**P < 0.01$.

Table S2. Primer sequences used for gene amplification in this study

Gene (for qRT-PCR)	Primer sequence (5'→3')
Fibronectin	Forward: TTA AGC TCA CAT GCC AGT GC<br>Reverse: CCC ACT TCT CTC CGA TCT TG
Vimentin	Forward: CTG CAC GAT GAA GAG ATC CAG<br>Reverse: ACT CGT TTG ACT CCT GCT TG
Igfbp5	Forward: ACG GCG AGC AAA CCA AGA TA<br>Reverse: GAG GGC TTA CAC TGC TTT CT
Galectin-1	Forward: AGC TTC AAT CAT GGC CTG TGG TC<br>Reverse: TCC CAG GTT CAG CAC AAA GCT C
Cxcl9	Forward: TCG GAC TTC ACT CCA ACA CAG<br>Reverse: AGG GTT CCT CGA ACT CCA CAC
Cxcl10	Forward: GAG AGA CAT CCC GAG CCA AC<br>Reverse: GGG ATC CCT TGA GTC CCA C
18sRNA	Forward: GCA ATT ATT CCC CAT GAA CG<br>Reverse: GGC CTC ACT AAA CCA TCC AA
